# Adulteration of Sports Supplements with Anabolic Steroids—From Innocent Athlete to Vicious Cheater

**DOI:** 10.3390/nu17193146

**Published:** 2025-10-01

**Authors:** Daniela Puscasiu, Corina Flangea, Daliborca Vlad, Roxana Popescu, Cristian Sebastian Vlad, Sorin Barac, Andreea Luciana Rata, Cristina Marina, Ionut Marcel Cobec, Sorina Maria Denisa Laitin

**Affiliations:** 1 Department of Cell and Molecular Biology, Faculty of Medicine, “Victor Babeș” University of Medicine and Pharmacy, 2nd Eftimie Murgu Square, 300041 Timisoara, Romania; puscasiu.daniela@umft.ro (D.P.); popescu.roxana@umft.ro (R.P.); 2 Department of Biochemistry and Pharmacology, Faculty of Medicine, “Victor Babeș” University of Medicine and Pharmacy, 2nd Eftimie Murgu Square, 300041 Timisoara, Romania; vlad.daliborca@umft.ro (D.V.); vlad.cristian@umft.ro (C.S.V.); cristina.marina@umft.ro (C.M.); 3 Toxicology and Molecular Biology Department, “Pius Brinzeu” County Emergency Hospital, Liviu Rebreanu Blvd 156, 300723 Timisoara, Romania; 4 Surgical Emergencies Department, “Victor Babeș” University of Medicine and Pharmacy, 2nd Eftimie Murgu Square, 300041 Timisoara, Romania; andreea.rata@umft.ro; 5 Vascular Surgery Clinic, Vascular and Endovascular Surgery Research Center, “Pius Brinzeu” County Emergency Hospital, Liviu Rebreanu Blvd 156, 300723 Timisoara, Romania; 6 Department of Obstetrics and Gynecology, Faculty of Medicine, Medical Center-University of Freiburg, 79106 Freiburg, Germany; 7 Clinic of Obstetrics and Gynecology, Klinikum Freudenstadt, 72250 Freudenstadt, Germany; 8 Department of Infectious Diseases, Faculty of Medicine, “Victor Babeș” University of Medicine and Pharmacy, 2nd Eftimie Murgu Square, 300041 Timisoara, Romania; laitin.sorina@umft.ro

**Keywords:** dietary supplement adulteration, testosterone-derivative compounds, identification methods, masking agents, doping, illicit use in sport

## Abstract

Some protein food supplements intended for athletes may be adulterated with pharmacologically active substances, including anabolic steroids and prohormones. The addition of these substances is aimed at enabling manufacturers to achieve rapid sales growth by promising quick increases in strength and muscle mass. However, the consumption of these products will lead to a positive result in a routine anti-doping test, along with all of the consequences that will directly affect an athlete’s career and reputation. At the same time, the illicit use of anabolic steroids continues to evolve across numerous sport disciplines. Moreover, vicious cheaters try to cover up their illegal actions by using various pharmacological agents to mask detection in anti-doping tests. This narrative review focuses on two situations—the innocent athlete and the vicious cheater. The athlete involved in inadvertent doping will suffer the consequences of doping, making close collaboration with medical staff extremely important. The analytic strategies described here address anabolic steroid doping detection and cheating using masking agents. This approach, based on biochemical changes, examines how these substances interfere with the testosterone pathway, from synthesis to elimination. Using masking agents alters the steroid profile, and the modifications produced by each agent are the subject of a detailed presentation. For most honest athletes, these findings support the initiation, development, and refinement of strategies for identifying food supplements with added illegal substances. Every athlete must have access to these approaches in order to avoid becoming vulnerable to sports fraud.

## 1. Introduction

Nutrient and vitamin deficiencies are a major problem in athletes because food intake alone becomes insufficient in the context of physical overload of the body. This deficiency must be corrected to maintain health and prevent illness, injury, and major exhaustion. The type of diet and dietary supplements (DS) selected, as well as the timing of DS use, are influenced by the sport practiced, the competitive period, the number of training sessions, and the athlete’s health status [1,2]. Also, in the presence of injuries, especially musculoskeletal ones, the use of DS with a high protein content/concentrated mixture of amino acids, creatine, omega-3, and antioxidants becomes imperative [3]. Moreover, when a viral infection occurs, such as SARS-CoV2 infection, vitamin and mineral supplementation becomes mandatory [4].

DS are used by most athletes in order to provide adequate nutritional intake for optimal performance. Often, the difference between an optimal athletic result and an exceptional one, as well as optimal recovery, is achieved through the combination of proper training and proper nutrition [5,6,7,8]. DS bring additional benefits, especially during prolonged exercise. The real therapeutic potential of these DS is sometimes overestimated, even though new regulations require the inclusion of the message “DS; contains ingredients which support physiological functions of the body by supplementing a typical diet; has no medicinal properties” [9]. Recently, there has been increased attention given to the adulteration of food supplements with pharmacologically active compounds that do not appear on the ingredients list. This practice is regulated by Commission Regulation (EU) 915/2023, which sets the minimum level of contaminants allowed [10]. Amidzic et al. presented an analysis of the Rapid Alert System for Food and Feed database regarding the illegal presence of compounds in DS, where, out of a total 474 substances, 16 were anabolics and prohormones [11]. Other data revealed that out of 248 sports supplements analyzed and detected with adulterants, 228 contained testosterone and other anabolic steroids [12,13].

Testosterone, a steroid hormone synthesized in the human body from cholesterol, has an important role in different stages of life. Starting from the structure of testosterone, a wide range of anabolic steroids has been synthesized over time. Both testosterone and testosterone analogs are therapeutic agents with a wide range of indications. They are predominantly used for their virilizing properties in men [14,15,16,17,18], anabolic effects in both men and women [14,19,20,21,22,23], and in some forms of breast cancer that are non-responsive to therapeutic action or resistant to cytostatic treatments [24,25,26,27,28]. Medical use involves much lower doses in standardized therapeutic protocols according to the indications of the corresponding pathology. In general, testosterone becomes attractive as a substance of abuse due to its anabolic properties, mainly through its ability to increase muscle mass and strength, along with the stimulation of erythropoiesis. Knowing the pharmacodynamic actions of testosterone and its derivatives, these compounds have been misappropriated from medical use to abuse in sports to increase athletic performance.

The use of testosterone began in the 1930s, with the isolation and observation of effects in humans, and proliferated in the Second World War, being used by soldiers to increase endurance and muscle strength [29]. From these findings, multitudes of alkylated derivatives were synthesized for oral administration.

In sports, at the beginning of the 1960s, athletes and bodybuilders began to use testosterone. At the Olympic Games of 1952 and 1956, athletes from the Democratic Republic of Germany and the Soviet Union used testosterone and its derivatives. The surprising results obtained by competitors (including women) in the 1960s and the appearance of these athletes drew attention to the abuse of anabolic steroids. Consequently, in 1974, the Olympic Committee decided to ban testosterone and anabolic steroids [30,31]. Since 1999, the World Anti-Doping Agency (WADA) has periodically generated lists of different prohibited substances that are (i) prohibited in all sports, both in competition and outside of it; (ii) prohibited only in competition; and (iii) prohibited only in certain sports [32,33]. The list currently in use is the one generated by WADA for the year 2025, where anabolic steroids are part of the category of substances prohibited under any circumstances [33]. Although there have been some changes in WADA regulations for some substances, such as marijuana-derived products [34], this consideration is far from being discussed for anabolic agents.

Inadvertent doping is a serious problem that affects both athletes and sports medicine practitioners. Along with development of the DS industry, which claims health benefits, especially for people who perform intense physical activity, there has been a growing tendency to use them less discerningly [35,36]. Several athletes have admitted to consuming various DS without seeking professional advice [37]. This behavior is not considered dangerous by them because DS are over-the-counter (OTC) medicines, but unfortunately, some are not properly controlled [12,38]. This behavior is common, especially in the context of preventing further damage, avoiding repetition of an unpleasant experience from the past, and managing stress, anxiety, depression, and the overall pressure placed on athletes [39,40]. In the case of a toxicological analysis, according to WADA regulations, full responsibility is directly assumed by the athlete [36]. Indirectly, the medical team involved is partially guilty in inadvertent doping situations [35,39].

From an ethical point of view, starting a sports competition under the influence of performance-enhancing substances is primarily contrary to the spirit of sports, and is clearly unfair. Furthermore, athletes are seen by society as role models in life, which, from a moral standpoint, is incongruent with doping. Achieving sports results through substance abuse is a method of cheating, banned both ethically and legally, especially in major sports competitions, where the prizes offered to the winners come with substantial financial rewards.

In this narrative review, we discuss a current and extremely hot topic, bringing to light the dual nature of dietary supplements: their real benefits and the potential risk of adulteration with anabolic steroids by the manufacturer. In many circumstances, involuntary use may improve sports performance. Conversely, vicious cheaters who abuse anabolic steroids may also attempt to mask this practice through various methods that can modify standard test results. However, in sports, the strategies and methods for masking doping often develop faster than detection techniques. In this narrative review, we highlight testosterone and anabolic steroid doping in sports, along with the strategies employed to conceal such doping. In addition, as methodology, we present toxicological strategies aimed at detecting anabolic steroids and masking agents, along with changes in the main metabolites of testosterone. An important point is the unintentional use of these steroids due to DS adulteration. We want to highlight these situations because there are innocent athletes who may pay the consequences, permanently impacting their career.

## 2. Methodology

The main objective of this narrative review is to emphasize the two extreme situations of positive anti-doping tests in sports: accidental (unintentional) doping, due to the consumption of dietary supplements by innocent athletes, and intentional abuse, where athletes consume anabolic steroids and employ various methods and strategies for masking their consumption (vicious cheaters). To achieve this goal, we discuss the currently existing detection methods and metabolic products or unmetabolized substances that are targets for identification according to the WADA criteria. For a better understanding, we include a description of the metabolic pathways of testosterone to explain the changes that occur under masking conditions. Finally, we discuss the main substances used as masking agents and the alterations they produce in identification profiles during anti-doping tests performed in major sports competitions. We used the PubMed, Google Scholar, and ScienceDirect databases, including review articles, original research, case reports, and meta-analyses, as well as the most recent WADA documents, among others, to clarify certain aspects related to doping with anabolic steroids. We excluded conference abstracts, letters to the editor, editorials, and comments that were not the subject of a peer-review process.

The keywords used were “testosterone”, “anabolic steroids”, “doping in sport”, “dietary supplements in sport”, “athletic personality”, “stable use”, “on-cycle”, “off-cycle”, “blast and cruise”, “athlete biological passport”, “WADA”, “epitestosterone”, “androsterone”, “etiocholanolone”, “5-a-androstane-3a,17-b-diol”, “dehydroepiandrosterone”, and their combinations. Other keywords used in writing the chapter dedicated to masking agents included compounds known in the field. These substance names are used as subchapter titles in this paper. We also considered standard analytical techniques, such as LC-MS, GC-MS, and IR-MS, in combination with the pharmacological agents mentioned above.

## 3. Synthesis and Metabolic Transformations of Testosterone and Major Synthetic Anabolic Steroids

The synthesis routes and main conversions of T presented in this chapter are intended to highlight certain intermediates that may be used voluntarily or accidentally for doping in sports, as well as the main structures that represent starting points for the laboratory synthesis of anabolic steroids. Furthermore, some transformations can be influenced by drugs in order to reduce the adverse effects of T and other steroids, or to mask detection in anti-doping tests.

**Figure 1:**
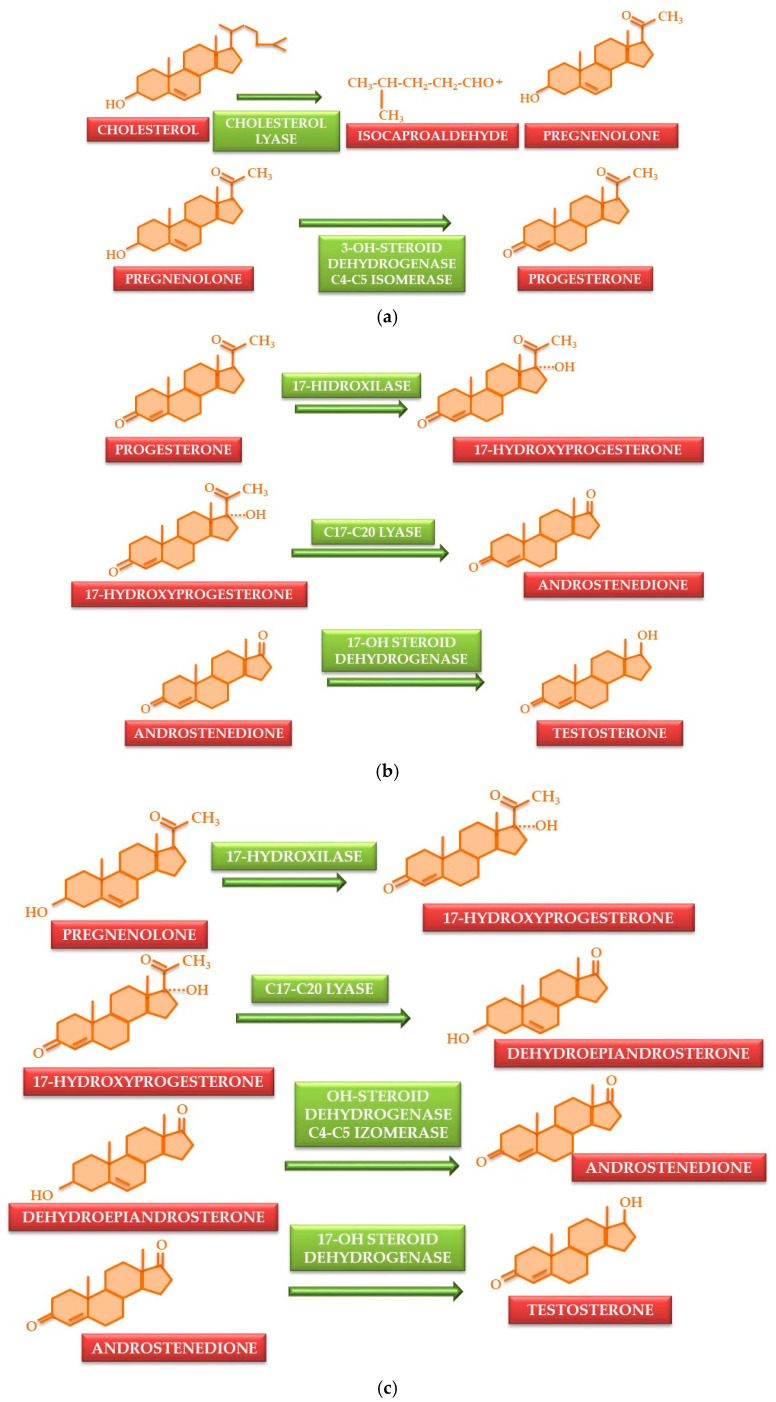
Testosterone synthesis pathway. The first step in biosynthesis from cholesterol is transformation to progesterone (**a**). Progesterone may be converted directly to testosterone via 17-hydroxyprogesterone (**b**) or by an intermediary step (**c**).

Testosterone is the principal androgenic hormone, synthesized from cholesterol, and characterized by a cyclopentanoperhydrophenanthrene nucleus containing 19 carbon atoms and lacking a side chain at carbon 17. It is produced predominantly by Leydig cells in men, accounting for over 90%, and to a lesser extent by ovaries in women and adrenal glands in both genders. Progesterone is derived from cholesterol and then follows two biosynthetic routes, one primary and one secondary [41,42]. Progesterone is synthesized initially from cholesterol through the cleavage of its side chain (Figure 1a).

The biosynthetic pathway from progesterone to testosterone diverges in two directions. The principal synthesis process involves the production of androstenedione through the action of the C17–C20 lyase enzyme on 17-hydroxyprogesterone, which is derived from progesterone via the action of 17-hydroxylase enzyme. Androstenedione is converted into testosterone by the enzyme 17-hydroxysteroid dehydrogenase (Figure 1b). A minor fraction of testosterone is synthesized from the conversion of androstenedione, involving an intermediary step where 17-hydroxyprogesterone is transformed into dehydroepiandrosterone, a reaction facilitated by C17–C20 lyase. Subsequently, dehydroepiandrosterone is converted into androstenedione, catalyzed by hydroxysteroid-dehydrogenase C4–C5 isomerase [42,43,44] (Figure 1c).

**Figure 2:**
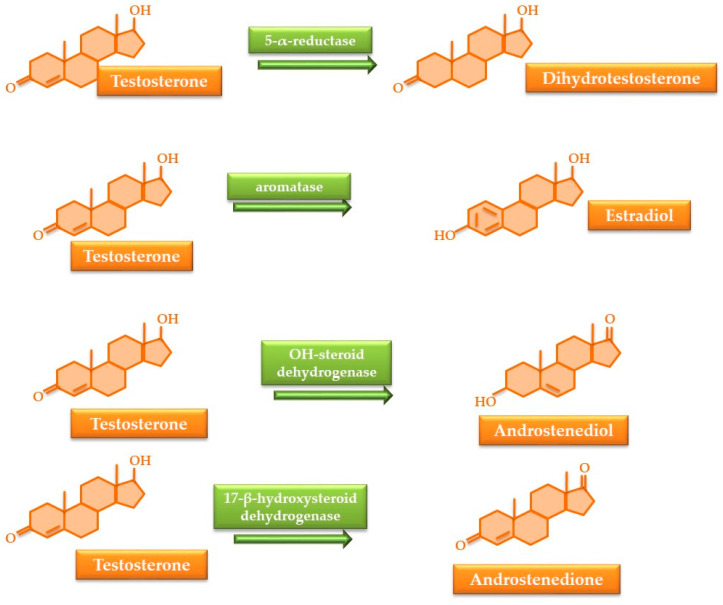
Minor changes in testosterone that become important under conditions of abuse.

Under physiological settings, a small percentage of testosterone can be converted percentage into dehydrotestosterone within the body, a process occurring in Leydig cells, the prostate, and seminal vesicles, resulting in particular androgenic effects. The enzyme implicated in this process is 5-α-reductase, which transforms roughly 8% of testosterone. Androstenediol can be synthesized in Leydig cells and in adrenal glands, producing androgenic effects. In the adrenal gland, 1% of testosterone can be converted into estradiol by aromatase, which plays a role in controlling hormonal output (Figure 2). In instances of abuse, these changes gain importance, and implicated enzymes will be targeted by drugs used as masking agents or to mitigate certain detrimental effects (e.g., gynecomastia) [32,44,45].

Testosterone has relatively low potency and is quickly metabolized by the liver; as a molecule, it is inactivated at the first hepatic passage, thus making its oral administration inappropriate. After intramuscular administration, it is fast absorbed and metabolized. It can be used structurally unchanged, in the form of a subcutaneous implant, transdermal patch, or transdermal gel provided with a pump [17,21].

**Figure 3:**
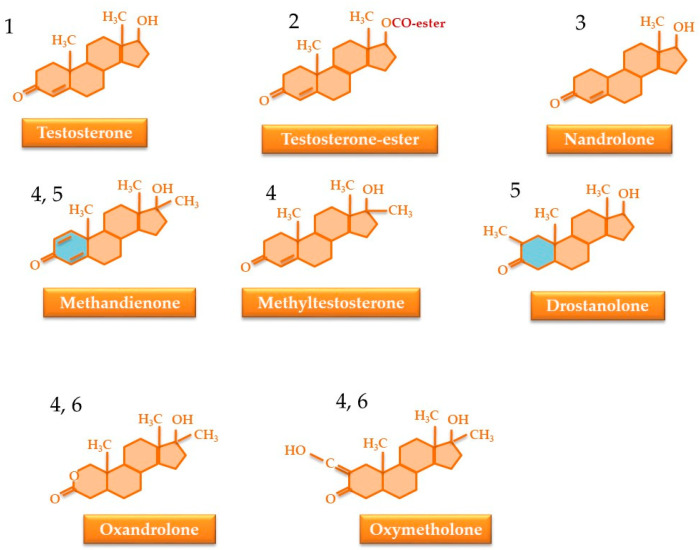
Modification of the testosterone structure in order to improve some properties or decrease side effects. (1) Structure of testosterone; (2) testosterone molecule esterification; (3) C1-demethylation; (4) C17 methylation or alkylation; (5) first-cycle modification; (6) loss of reduction capacity of testosterone.

To reduce these drawbacks, the testosterone molecule has undergone numerous changes that confer different advantages. Currently, there are over 100 synthetic derivatives on the market both from the laboratories of pharmaceutical companies and from illicit laboratories. These modifications are as follows [46,47,48] (Figure 3):

- Esterification at the 17-OH group causes a reduction in release speed, yielding formulations with sustained release. Esterified testosterone forms, such as undecanoate, propionate, phenylpropionate, enanthate, cypionate, and undecylate, used in form of an oily solution, exhibit slower absorption and prolonged action due to the gradual enzymatic hydrolysis of the ester bond at the site of administration [49,50].
- C10 demethylation increases the relative potency of substances. These compounds can also be C17 esters; e.g., nandrolone [51].
- C17 methylation/alkylation reduces the rate of metabolism during the first hepatic passage, allowing the substance to be administered orally; e.g., methandienone, methyltestosterone [47,52].
- Modifications of the first cycle prevent the substance from functioning as a substrate for aromatase. Thus, they are not transformed into estradiol and have no androgenic effects, e.g., methandienone, drostanolone [53,54].
- Androgens that cannot be reduced to dihydrotestosterone exhibit a ratio of anabolic to androgenic actions that favors anabolic effects, e.g., oxandrolone and oxymetholone [55].

These transformations are important when identifying testosterone, prohormones, and other anabolic steroids in a routine anti-doping test.

## 4. Innocent Athlete: Adulteration of Dietary Supplements with Testosterone, Testosterone Derivatives, and Other Synthetic Anabolic Steroids

The issue of DS adulteration involves both athletes and the manufacturing companies. In the following section, we present the possible justification of DS manufacturers for adulteration, studies that identify anabolic steroids in DS, the repercussions for athletes, and some suggestions for avoiding unintentional consumption of these types of DS.

Introduction: Adulteration of dietary supplements for athletes is a serious problem in major sporting competitions and beyond. Widely known protein supplements convey the message, in one form or another, that they are responsible for increasing muscle mass. Protein-based DS show benefits in terms of recovery and increasing muscle strength and mass [56,57,58]. Several studies have demonstrated that reality does not always correspond exactly to what they claim [59,60].

Motivation for adulteration: The introduction of pharmacologically active adulterants, such as anabolic steroids and prohormones, aims to increase sales of these products by removing discrepancies between what is desired (rapid, visible increase in muscle mass and strength) and what actually occurs (additional protein intake makes a visible contribution after years of use in combination with consistent, well-organized training). Anabolic steroid chemicals are illicitly incorporated into various protein supplements for athletes, and the manufacturers do not declare them in the description of the product’s composition listed on the packaging [36,61,62,63,64].

Identification of substances: In some studies, 54 [61], 67 [62], and 93 [65] of such substances were identified in protein supplements. Moreover, 52.2% of DS investigated in one study showed contamination with anabolic steroids [66], while in other study, 25 of 66 DS contained prohibited substances [37]. These adulterations cause a positive test in athletes who consume DS without the intention of doping or cheating, and usually are accompanied by a multitude of adverse effects [58,67,68]. In some situations, prohormones and other steroids, including DHEA, 4-androstenedione, 4-androstenediol, and 5-androstenediol [67], are sold as DS, and some of them come from the black market [69].

Consequences for athletes: The consumption of these substances will lead to their identification in routine anti-doping tests. Athletes should know that they are judged based on their anti-doping test results, regardless of whether they acted intentionally or unintentionally. Since ignorance is no excuse, they must be informed on this fact [35]. Another problem is prescribed medications, such as anxiolytics and antidepressants, which involves collaboration between medical staff and other specialists, such as psychiatrists, to maintain a proper state of health in athletes without jeopardizing the results of anti-doping tests [39]. Even if utilization of these substances is not intentional, the athlete will suffer the consequences, which will greatly affect their sports career.

Potential solutions: Currently, portable methods are being developed in order to identify the authenticity and adulteration of protein supplements, in particular whey protein supplements [70,71,72]. Among the recommended methods for identifying DS adulteration, considered superior to the classic high-performance liquid chromatography (HPLC) technique, are capillary electrophoresis (CE) [73], ultra-HPLC coupled with quadrupole time-of-flight mass spectrometry (UHPLC-QTOF-MS) with all-in fragmentation acquisition [74], or using surface-enhanced Raman spectroscopy (SERS) [75]. Subjecting DS to such analyses is recommended before their use, especially if the athlete is preparing for or participating in an important competition.

## 5. Non-Therapeutic Patterns of Steroid Administration in Sport

The use of steroids in sports always involves some degree of addiction, influenced by social and psychological factors. Additionally, certain schemes are established empirically by athletes, without having scientific justification. For those in their entourage, these schemes are easily recognizable and, over time, undergo variations depending on the athlete and type of anabolic steroids available on the market. These aspects are presented below.

Administration patterns and dependence: Non-medical administration of testosterone and its derivatives do not follow the known therapeutic schemes; instead, there are specific models that athletes count on to deliver the desired benefits, including delaying tolerance, reducing some side effects, and decreasing the possibility of detection. Analysis of data from multiple studies [76] involving athletes, particularly bodybuilders and weightlifters, revealed that testosterone and its derivatives induce dependence, particularly among individuals who utilize high doses and combine numerous androgenic substances without adequate steroid-free intervals. This characteristic is sustained and intensified by muscular dysmorphia, a condition prevalent among a significant proportion of anabolic steroid users [77].

Psychological factors and social pressure: Aside from physical appearance, other psychological issues contributing to the utilization of testosterone and its derivatives are encompassed within the concept of “athletic personality.” Among them are perfectionism and athletic identity, which can manifest positively, aimed at succeeding optimal achievements, or negatively, focused on evading failure. Both good and negative inclinations may be linked to steroid usage [78]. These issues are prevalent in both genders; nevertheless, a preference for oral administration is noted exclusively among women [79]. Social pressure also has a major influence on both sexes in terms of steroid abuse [78,79].

The cyclical model: There are several administration patterns described. “Stable use” refers to continuous use of the same dose. This model is currently less common [76]. The predominant approach is “cycling” use, where consumption periods of 6-12 weeks called the “on cycle” alternate with longer periods called the “off cycle” during which the individual abstains from anabolic steroids entirely [80,81].

Post-cycle therapy: During the “off cycle” period, athletes may consume substances that limit the adverse effects of testosterone and analogs and enhance the recovery of hypothalamic–pituitary–gonadal axis function [45,80]. This phase, called “post-cycle” therapy, involves self-administration of (i) human chorionic gonadotropin (HCG) to restore endogenous testosterone secretion; (ii) finasteride and other α-reductase inhibitors that reduce the amount of testosterone transformed into dihydrotestosterone (Figure 2); (iii) anastrozole and other aromatase inhibitors that reduce the transformation of testosterone into estradiol (Figure 2); (iv) tamoxifen and other antiestrogens that, together with aromatase inhibitors, reduce or prevent gynecomastia [80,81,82,83].

The ”blast and cruise” model and staking: “Blast and cruise” administration is a variant of “cycling”, where high doses of anabolic steroids are used with a longer period (“blast”), followed by shorter periods (“cruise”) in which a maintenance dose with small amounts of the substance is used in order to prevent muscle atrophy. Unlike the classic “cycling” form, the “blast and cruise” version does not include complete cessation of anabolic steroid [65,80,84]. Usually, in these schemes, anabolic steroids are “stacked”, which is the association of several anabolic steroids in small doses to reduce adverse reactions, leading to a desired cumulative effect [81]. This method of consumption starts from the idea that small doses of different compounds can reduce undesirable events compared to a higher dose of a single compound. In this regime, supraphysiological doses are administered over long periods to minimize the plateau effect [82].

## 6. Detection of Abuse in Laboratory

Anti-doping tests in sports are an integral part of major competitions. They involve various analytical techniques that detect, primarily in urine but also in other biological matrices, substances of abuse and their metabolites. Sometimes, doping can be suspected based on physical appearance and changes in routine laboratory analyses. To present methodologies used in anti-doping detection, and, in order to better explain quantified metabolites and notable transformations in the case of abuse, the T degradation pathway in the body is introduced. This description is the starting point of this chapter, where we highlight the transformations detected in situations of attempted use of masking agents.

Physical and behavioral signs of abuse: Difficulties in recognizing athletes who misuse anabolic steroids to enhance their sports performance are significant, particularly in prestigious tournaments like the European and World Championships, the Olympic Games, or other events that offer substantial financial rewards for winners. Attention is paid especially to competitors who display certain visible physical signs associated with the consumption of anabolic steroids. Thus, the presence of acne, gynecomastia, skin striae, and signs of needle pricks in the election areas for intramuscular administration, raises the suspicion of illicit use of anabolic substances. Regarding women, the signs include general appearance (taking on a masculine characteristics), the presence of hirsutism, atrophy of the breasts, and deepening of the voice [85,86]. In addition, upon careful observation, certain behavioral disorders are visible in these people in terms of aggressive behavior, with noticeable psychological and cognitive problems, as well as anger management in general [86,87].

Hematological and hepatic alterations: There are also some changes in laboratory analyses routinely performed before important sports competitions, such as increases in the number of red blood cells and hemoglobin levels [88,89]. This impact results from activation of bone marrow progenitor cells; the erythrocyte lineage responds first, evidenced by an increase in the red blood cell count, followed by neutrophils, and subsequently, the thrombocyte lineage with an increased platelet count [90,91]. Liver enzymes such as alanine aminotransferase (ALT), aspartate aminotransferase (AST), alkaline phosphatase (ALP), creatine kinase (CK), lactate dehydrogenase (LDH), and gamma glutamyl transferase (GGT) will exhibit alterations, particularly following the administration of C17-alkylated compounds due to their heightened hepatotoxicity and augmented risk of cholestatic jaundice development [92,93]. Additionally, elevated levels of LDL-cholesterol and blood glucose may occur concurrently with a reduction in HDL-cholesterol [94].

Detection challenges and the athlete biological passport: In general, detection is quite difficult to perform. Athletes utilize anabolic steroids during training to complicate detection, abstaining from their use during competitions. The detection window for oral preparations ranges from 2 to 14 days, whereas for injectables, it extends to 4 weeks. After these intervals post-withdrawal, detection is difficult [95]. The implementation of the Athlete Biological Passport (ABP) by WADA aimed to detect athletes whose athletic performance is attributable to substance usage rather than their inherent capabilities. With the help of ABP, the aim is to quickly identify the use of illegal substances, discourage this use, and manage the prevention of fraud [96]. However, a lot of discussions have emerged over its validity because it is an imperfect model that seeks to make a clear distinction between doping and certain individual characteristics [94,97,98]. Nowadays, it is necessary to introduce artificial intelligence to process all these variables through specific algorithms that can accurately identify suspected cases of doping, determine their authenticity, and establish which cases should be especially followed up over time [96].

The steroid module and the T/E ratio: The current WADA technical documents [99,100,101,102] comprise three modules: hematological, steroidal, and endocrine. The steroidal module contains several urine determinations measuring both free and glucuronide fractions, the latter requiring release by hydrolysis. The classic method for detecting testosterone and its derivatives is the determination of the T/E ratio in urine. This method is based on the fact that testosterone (T) and its 17-α-epimer, epitestosterone (E), are synthesized at a 1:1 molar ratio. Physiologically, the T/E ratio is 1; increasing this ratio is produced only by using exogenous testosterone [95]. Because there are individual variations and to highlight attempts to mask illegal use in sports, WADA enforces the use of several tests in the ABP [99,100,101,102]. Table 1 presents the lab analyses performed by chromatographic methods, the urinary concentrations beyond which doping is suspected, and the ratios between different compounds. In this assessment, steroidogenesis enhanced by intense physical exertion during the competitive season must also be taken into account [103,104].

**Figure 4:**
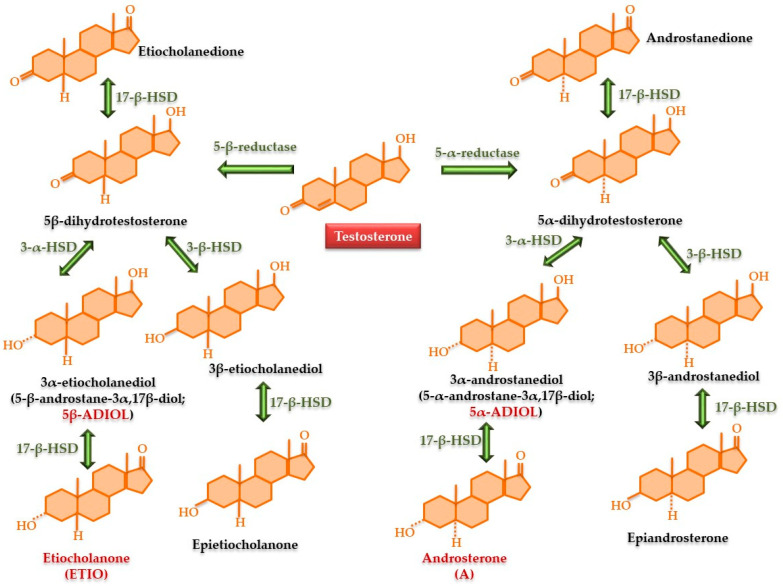
The metabolic pathways of testosterone transformation into 17 ketosteroids with practical importance in the detection of illegal use in sports. 3α-HSD = 3α-hydroxysteroid dehydrogenase; 3β-HSD = 3β-hydroxysteroid dehydrogenase; 17β-HSD = 17β-hydroxysteroid dehydrogenase.

The metabolic pathway of testosterone: The steroid profile module includes 17-ketosteroid metabolites of testosterone. They are synthesized from testosterone through the action of α and β reductases to produce 5-α- and 5-β-dihydrotestosterone (DHT). 17-β-hydroxysteroid dehydrogenase (17-β-HDS) converts the 17-OH group into a keto group, yielding androstanedione and etiocholanedione. When 3-α-HDS or 3-β-HDS interacts with α- or β-DHT, the 3-keto site undergoes hydrogenation to form an OH group, yielding two isomers: 3-α-androstanediol and 3-β-androstanediol. Likewise, the enzyme’s activity on 5-β-DHT will saturate the 3-keto group at the hydroxyl (OH) position, resulting in the formation of 3-α- and 3-β-ethiocholanediol. In the last stage, the enzyme 17-β-HDS modifies all these compounds, converting the 17-OH group into 17-keto, yielding androsterone, epiandrosterone, etiocholanone, and epietiocholanone [105,106,107]. According to WADA recommendations [99,100,101,102], testosterone and its isomer epitestosterone (E), synthesized in a 1:1 molar ratio, along with androsterone (A), etiocholanone (ETIO), 3-α-androstanediol (5-α-androstane-3α-,17-β-diol or 5-α-ADIOL), and 3-α-etiocholanediol (5-β-androstane-3α-,17-β-diol or 5-β-ADIOL), are diagnostically significant, as are their ratios [108] (Figure 4).

Chromatographic and spectrometric analytical methods: In addition to testosterone and its metabolic derivatives, the WADA recommendations [99,100,101,102] encompass the identification of synthetic steroids and their equivalents. Contemporary analytical approaches, such as MS techniques integrated with various chromatographic systems, may identify steroid-type synthetic chemicals, their metabolites, and diverse artisanal anabolic drugs from illicit laboratories [62,63,64,109,110,111]. No matter how complex these molecules are, the gas chromatography-MS (GC-MS) [112,113,114], LC-MS [115,116,117,118], and immunoaffinity chromatography-MS (IAC-MS) techniques [119,120] can successfully meet this challenge. This has been demonstrated by various studies, where simple lipids (fatty acids, sterols) [121,122,123,124,125,126], complex lipids (triglycerides, phospholipids) [127,128,129], and ultra-complex lipids (gangliosides, various sphingolipids) [130,131,132] could be detected in quantities up to nanogram/mL.

Isotope ratio mass spectrometry technique: Another analysis method used to detect the illegal use of testosterone in sports and included in the methods for ABP is isotope ratio-MS (IR-MS) [133]. IR-MS is particularly indicated where there are suspicions of testosterone abuse, especially when the T/E ratio is at the upper limit, as it is necessary to clarify the exogenous origin of testosterone. Carbon isotope ratio ^13^C/^12^C identifies the origin of testosterone, with^13^C indicating an exogenous source and ^12^C indicating an endogenous source [46,120,134]. In general, cholesterol can be synthesized endogenously or brought into the body through food intake depending on the type of diet. This cholesterol will be converted into androgen hormones. The characteristics of cholesterol-derived compounds from plants differ depending on the natural processes of CO_2_ incorporation. C3 plants (wheat, soybean, rice) incorporate CO_2_ from monosaccharides with five carbon atoms, resulting in intermediates formed out of three carbon atoms. The key enzyme of this process, ribulose-1,5-diphosphate, has a higher affinity for ^12^C compared to ^13^C, so these plants will have a higher content of ^12^C [135,136]. C4 plants (maize) attach CO_2_ to phosphoenolpyruvate, resulting in intermediates with four carbon atoms. Phosphoenolpyruvate carboxylase, the key enzyme of the process, has no special affinities for ^12^CO_2_ or ^13^CO_2_. This pathway has a lower impact on the ^13^C/^12^C ratio compared to the pathway, followed by C3 plants [137,138]. There are also plants that operate with a metabolic combination of both pathways (pineapple), called Crassulacean Acid Metabolism (CAM) plants, which produce a higher ^13^C/^12^C ratio than C3 plants [139]. Most pharmaceutical companies perform a semisynthesis of steroids, often commencing with diosgenin from soybeans [140]. In this way, exogenous steroids contain a higher amount of ^12^C. The ^13^C/^12^C ratio is significantly reduced in the case of exogenous administration of sterol derivatives compared to those synthesized endogenously from dietary cholesterol. Since dietary intake is an important factor influencing the isotopic ratio, in order to exclude this interference and highlight only exogenous anabolic steroids, calculations refer to Endogenous Reference Compounds (ERCs). The value is expressed as δ^13^C and the result is an isotopic difference Δ between the sample and the ERC, where ^13^C/^12^C standard represents the international Vienna Pee Dee Belemnite (VPDB) standard. [109,124,141,142].

Δ (‰) = δ^13^C_ERC_−δ^13^C_sample_

δ13C=(C13/Csample12C13/CVPDB standard12−1)×1000

δ^13^C is considered to have values of −22‰ for a typical European diet and −17‰ for a C4-based diet [143]. Without exogenous anabolic steroid administration, the Δ‰ values of metabolites and precursors are close. A value greater than 3‰ is considered indicative of exogenous steroid use [142].

For this test, the ^13^C/^12^C cholesterol ratio in the same person is used as an internal standard for each determination [144]. The isotopic ratio can also be applied for testosterone metabolites, as illustrated in Table 1 and Figure 4 [145,146,147,148]. Other endogenous steroid derivatives and their metabolites, such as adrenosterone, 11-β-hydroxy-androsterone, 11-β-hydroxy-etiocholanone, 11-oxo-androsterone, and 11-oxo-etiocholanone, which are on the WADA prohibited list, can also be identified and quantified by the carbon isotope ratio [109].

Detection limits and interfering factors: In general, GC-MS analysis is a classic, rapid method that provides qualitative and quantitative analysis of a large number of anabolic steroids and their metabolites, but it can be laborious and slow because it requires derivatization [112] or protectants such as polyethylene glycol 400 to improve the signal [149]. LC-MS-based methods do not require derivatization and can identify different compounds and metabolites in complex mixtures, especially in the context of consuming anabolic steroids originating from illicit laboratories [150,151]. In general, both techniques are routinely used in a complementary manner, LC-MS also being able to identify glucuronide metabolites resistant to hydrolysis [152,153]. In general, a negative result in GC-MS analysis is followed by LC-MS investigation [153]. The detection limits of steroids in urine were 100 pg/mL for stanozolol and metabolites using GC-MS [154], and 2–100 pg/mL with a quantitation limit of 0.05–2 ng/mL using GC-capillary photoionization tandem MS [155]. UHPLC-MS showed a lower quantification limit of 5–1000 pg/mL in serum [156], while GC-combustion-IR-MS indicated a mean δ^13^C range of 3‰–3.3‰ for androstenedione, testosterone, and dehydroepiandrosterone [157]. A study where a single dose of 80 mg testosterone undecanoate was administered orally, followed by a second 80 mg dose 48 h later, demonstrated that detection in urine by GC-MS had quantification limits of 0.5 ng/mL for T, 1 ng/mL for E, 2 ng/mL for 5-α-ADIOL and 5-β-ADIOL, and 50 ng/mL for A and ETIO. After 48 h, when plasma T could no longer be detected by UHPLC, mean urinary δ^13^C values corresponding to 0.11 ‰ for T, 0.17‰ for A, 1.12‰ for ETIO, 1.6‰ for 5-α-ADIOL, and 2.1‰ for 5-β-ADIOL were evidenced [158]. Simultaneous determination of these parameters by two different methods, one of them being IR-MS, increases the chances of detection for individuals with a T/E ratio <6, an aspect also highlighted by other authors [158,159]. All these analyses must be performed in the absence of urinary bacterial flora due to the alteration of the results through deconjugation of A and ETIO by bacterial enzymes and intervention of a microbial 3-hydroxysteroid-dehydrogenase [107,160]. Alcohol can also induce false positive results by increasing urinary elimination of T-glucuronide, raising DHEA production, and lowering elimination of A-glucuronide, resulting in an increase in the T/E ratio and a decrease in the A/T ratio [107,161].

Alternative analyses—the potential of hair: These parameters are usually carried out using urine samples. To increase the detection window to months or even years, some studies have shown that identification of anabolic steroids can be performed successfully using hair [162,163,164]. Laboratory detection from hairs belonging to various areas of the body (head, arm, leg, chest, pubic area) evidenced the presence of anabolic steroids [165,166]. Moreover, in a comparative study that analyzed hair from different parts, a higher concentration and a larger detection window was observed in hair collected from other areas of the body compared to scalp hair [166]. Even though there is a large number of studies that certify the practical usefulness and reliability of these determinations, currently, WADA does not accept hair as a biological sample for detection of anabolic steroid abuse [99,100,101,102,164].

## 7. Vicious Cheater Athletes: Pharmacological Agents Used to Mask Identification

A whole series of strategies have been approached by people who use anabolic steroids in sports, often to cover up the identification and to reduce some of the adverse effects, especially those related to physical appearance. For this purpose, in addition to screening for testosterone, metabolites and derived steroids, WADA also enforces screening for the main substances used as masking agents [99,100,101,102].

Dihydrotestosterone (DHT) is one of the main androgens with a higher potency than testosterone. It is used illegally as an alternative to T, being a downstream metabolite of T [107,167]. DHT does not influence the T/E ratio because the reaction catalyzed by 5α-reductase is irreversible. Both T and DHT bind to the same androgen receptor and produce similar, tissue-dependent effects [168]. DHT is used instead of T as a cheating method, where detection involves only evaluating the T/E ratio. Administered exogenously, it causes an increase in the 5-α-ADIOL/E and A/E ratios, intensifying the elimination of DHT metabolites. The metabolism of exogenous DHT will follow α degradation pathway because 3-α-HSD, 3-β-HSD, and 17-β-HSD have a higher affinity for 5-α-DHT than for 5-β-DHT (Figure 4). Consequently, exogenous intake of DHT will favor preferential increases in the metabolites androsterone, androstanedione, and 5-α-ADIOL, with an elevation in the 5-α-ADIOL/5-β-ADIOL ratio [107,169,170].

Dehydroepiandrosterone (DHEA), a substance that can also be purchased online, has often been used as an alternative to T [171]. DHEA is an upstream precursor of T and is used in place of T for its anabolic-like effect. Molecularly, DHEA yields both E and T [172] and is used illegally without detection, where anti-doping tests only monitor the T/E ratio. It has been shown that, in the case of DHEA use, its concentration is not significantly increased in urine, but its concentrations of metabolites 5-α-ADIOL and 5-β-ADIOL (both included in ABP) are raised significantly. Being a precursor in testosterone synthesis (Figure 1c), following metabolism, the T/E ratio does not change (both testosterone and epitestosterone concentrations are increased in urine). By transforming DHEA into testosterone, the urinary concentrations of all downstream metabolites will increase, including ETIO, 5-α-ADIOL, and 5-β-ADIOL, as well as those specific for DHEA, including 7-keto-DHEA, 7-β-hydroxy-DHEA, and 3-α-5cyclo-DHEA [173,174,175]. However, studies have evidenced that the short-term use of DHEA does not significantly increase athletic performance, but its metabolites will be identified in the urine starting 24 h after the last cessation [176,177].

Androstanedione is a downstream intermediate in testosterone metabolism (Figure 4). Androstanedione is converted into DHT, and its illegal administration results in elevated metabolite concentrations in a manner similar to illicit the administration of DHT [107,178].

Epitestosterone (E) is illicitly used together with testosterone to enable athletes to mask detection methods that rely only on the T/E ratio [179]. Quantitative determination of T, E, and their metabolites according to WADA recommendations (Table 1) [99,100,101,102], along with analysis of the ^13^C/^12^C isotopic ratio [46,120,133,134,145,146,147,148], identifies doping and attempts to mask it.

Human chorionic gonadotropin (HCG), known as the pregnancy test hormone, is one of the compounds on the WADA list of prohibited substances [33]. It is used to stimulate testosterone production and restore gonadal function after suppression caused by anabolic steroid use. HCG can be detected in urine by immunoassay methods even 7–10 days after administration. A positive result in men is considered if the value exceeds 5 IU/L [180]. Furthermore, the use of HCG to stimulate testosterone production will cause a simultaneous increase in the excretion of testosterone and epitestosterone without influencing the T/E ratio. In general, in a routine urine analysis for the detection of steroid abuse, no change will be observed outside of the parameters presented in Table 1. In this case, only the express detection of HCG highlights the illicit use [181].

Luteinizing hormone (LH) is another compound capable of increasing endogenous testosterone production in men, belonging to the list of substances prohibited in sports [33]. As with HCG, the use of LH does not significantly alter the T/E ratio. In addition to the identification of LH by immunoassay methods, several studies [181,182,183] have evidenced that evaluation of the T/LH ratio can be a reliable indicator for the use of HCG as an illicit method of stimulating testosterone production. A T/LH ratio value above 30 is considered indicative of this. Determination of the T/LH ratio is taken into account by WADA [184].

α-reductase inhibitors (finasteride, dutasteride) are drugs used for the treatment of benign prostatic hypertrophy [185,186,187]. These drugs selectively inhibit the conversion of testosterone to 5-α-DHT, causing a decrease in the concentration of metabolites located downstream of the inhibited enzyme, especially A and 5a-ADIOL (Figure 4). By reducing the urinary concentrations of A and 5-α-ADIOL, there will be slight increases in 5βADIOL, ETIO, and the 5-α-ADIOL/5-β-ADIOL ratio, while the 5-α-ADIOL/E ratio will significantly decrease, without significantly modifying the T/E ratio (Table 1) [188,189]. Administration of finasteride (5 mg/day) together with T enanthate (125 mg/week), causes an increase in T and estradiol concentrations with a concomitant reduction of DHT, contributing to improvements in performance, muscle mass, and strength [190,191]. In addition, finasteride combined with T increases T bioavailability and the free circulating fraction by reducing the sex hormone binding globulin [190]. When combining 5 mg finasteride with 19-norandrostenedione, the main metabolite of 19-norandrostenedione, norandrosterone, presented a urinary decrease of up to 84%, causing an alteration of the steroid profile and masking the use of illegal steroids by generating false negative results [192]. Along with these changes that can mask the identification of steroid abuse in sports, routine screening by chromatographic methods coupled with mass spectrometry can identify the presence of finasteride, dunasteride, and their hydroxylated metabolites [193,194,195].

Aromatase inhibitors (anastrozole, letrozole, exemestane) are drugs used in the treatment of some forms of estrogen-dependent breast cancer [196,197,198]. They are not used to mask detection. Instead, aromatase inhibitors are abused by male athletes to avoid gynecomastia, as well as effects produced by estrogen accumulation. They inhibit the irreversible conversion of testosterone to estradiol by removing the C19 methyl group and aromatizing the first cycle (Figure 2). These aromatase inhibitors can be identified both in urine and hair by LC-MS screening [199,200].

Antioestrogens (tamoxifen, clomiphene, raloxifene) are drugs that, similar to aromatase inhibitors, are not used to mask detection but to reduce estrogenic side effects. Therapeutically, they are used for the treatment of metastatic breast cancer that expresses estrogen receptors [201,202,203,204,205,206]. They can be administered “post-cycle”, especially by bodybuilders, in combination with aromatase inhibitors following different protocols described by Rochoy et al. [199]. Briefly, these protocols extend over a period of 21 days. The first protocol begins with daily administration of 250 IU HCG for 3 days, followed by the administration of both clomiphene and tamoxifen in decreasing doses. The other protocol is not preceded by the use of HCG but combines exemestane with the concomitant use of clomiphene and tamoxifen. Another study [207] highlights an increase in serum testosterone and gonadotropin concentrations after a 30-day course of clomiphene. The study group was formed by male athletes between 25 and 38 years old who used recreational testosterone derivatives, receiving 1 tablet of 50 mg/day during this entire 30-day period. Another recent paper [208] demonstrated that clomiphene can be used off-label for the treatment of anabolic steroid-induced hypogonadism due to its property of stimulating endogenous testosterone production.

Probenecid is a uricosuric agent used in the treatment of gout that acts by inhibiting tubular reabsorption of uric acid and promoting its elimination [209,210,211]. Probenecid also has a competitive inhibitory effect on tubular secretion, hindering the elimination of certain drugs excreted mainly in the form of glucuronide conjugated metabolites, such as penicillin [212,213,214]; similarly, probenecid decreases the urinary excretion of steroids excreted as glucuronidated metabolites [215]. Probenecid affects anabolic steroids in two aspects: (i) it reduces their urinary elimination by decreasing the urinary concentration below the detection limit and preventing identification in routine urinalysis; (ii) it reduces urinary elimination, maintaining the plasma concentration of steroids and their derivatives for a longer time at pharmacologically active concentrations [107,216]. Upon closer screening of urine, probenecid can be identified by GC-MS methods [217]. On the other hand, a recent study [218] demonstrated an increase in the release of Ca^2+^ from intracellular stores at rest, causing an increase in resting voltage. The consequence of this effect would be a reduction in muscle strength, discouraging the use of probenecid to enhance athletic performance.

Diuretics increase water excretion and urine volume with the dilution of steroids and their metabolites. The use of diuretics will only alter the urinary concentration without affecting the ratios between testosterone and its metabolites, as illustrated in Table 1 [107]. Some diuretics cause a decrease in urine specific gravity (thiazide diuretics, loop diuretics), and others produce a urinary alkaline pH reaction (carbonic anhydrase inhibitors such as acetazolamide) [219]. Before GC-MS and/or LC-MS routine urine screening for the detection of doping in sports, according to WADA recommendations [99,100,101,102], the determination of specific gravity and pH must be performed. A study using improved technology was able to detect 50 diuretics and other masking agents by solid phase extraction combined with LC-MS analysis [220]. A recent study highlighted that 19.5% of professional soccer athletes from Brazil used diuretics and various masking agents, while 15% reported the use of anabolic steroids during the national championship (48.7% of these athletes tested positive for doping substances, with the majority—31%—consuming stimulants) [221]. There has also been a reported case of a 28-year-old male bodybuilder who died after consuming a mix of anabolic steroids, human growth hormone, and thyroid hormone while training for a competition. He had taken furosemide 24 to 48 h prior to the competition. Death occurred due to hypokalemic paralysis and rhabdomyolysis at the end of the competition [222].

Azole-derivatives (ketoconazole, miconazole) are drugs used in the treatment of fungal infections. This effect is due to the inhibition of ergosterol synthesis by acting on the enzyme lanosterol demethylase. The accumulation of ergosterol biosynthesis intermediates activates C5,6 desaturase, converting them into methylated compounds toxic to fungal cells [223]. In addition, ketoconazole is also used for the treatment of endogenous Cushing’s syndrome in adults and adolescents over 12 years old because ketoconazole is a hormonal production inhibitor of the adrenal gland. Ketoconazole inhibits 17-hydroxylase and the 11-hydroxylation processes, and at high doses, also acts on the cholesterol side-chain cleavage enzyme. Therefore, ketoconazole is an inhibitor of cortisol and aldosterone synthesis. Ketoconazole also acts as an inhibitor on androgen production by inhibiting the activity of C17–20 lyase in the adrenal gland and Leydig cells [224] (Figure 1). Due to this mechanism, ketoconazole can be used as a masking agent for anabolic steroids in sports, with reductions in the urinary elimination of testosterone, A, ETIO, and the T/E ratio [225]. Based on these findings, in a trial involving the administration of miconazole for one week at 500 mg/day dose to healthy volunteers consuming testosterone derivatives, decreases in urinary concentrations of A, ETIO, and the A/T and A/ETIO ratios were observed [226]. In another study where miconazole was administered, decreases in the urinary concentrations of A, ETIO, 5-α-ADIOL, 5-β-ADIOL, and the A/ETIO, A/T, and 5-α-ADIOL/E ratios were also observed, but with an increase in the 5-α-ADIOL/5-β-ADIOL ratio [226]. Due to these findings, WADA also recommends screening for this category of substances [99,100,101,102]. For clarity, Table 2 schematically displays these masking agents.

Perspectives on alternative biomarkers: In the context of rapid development of the illicit anabolic steroid industry, along with the numerous alternatives for masking detection described above, it is necessary to find biomarkers that are not influenced by masking techniques. One possible approach would be to identify the fingerprint left by anabolic steroids on microRNAs (miRNAs). When there is damage to different organs, many types of miRNAs are released into circulation [153,227]. Anabolic steroids exert a lasting impact on the heart, brain, muscular system, liver, and kidneys [227,228]. For example, in the case of cocaine abuse, increases in the expressions of brain miRNA-132, miRNA-144, and miRNA-34 are observed [228,229], and miRNA-132 and miRNA-144 are also associated with anabolic steroid use [229]. At the renal level, miRNA-21 and miRNA-205 expression were elevated in individuals who abused anabolic steroids [230]. Another study highlighted increased expression in renal-derived miRNA-146a after nandrolone administration in Wistar rats [231]. Further research is needed to reveal a specific miRNA pattern for the abuse of different types of anabolic steroids that will probably pose fewer problems in terms of abuse masking strategies.

Another possibility is the analysis of enzyme polymorphisms involved in steroid metabolism. In the case of UGT2B17 deletion, a mutation that reduces the glucuronidation of T and metabolites, with a decrease in their urinary elimination, was studied [232]. It was observed that the most reliable parameters were the androstanediol sulfate/dehydroandrosterone sulfate ratio and the epiandrosterone sulfate/dehydroandrosterone sulfate ratio [233]. Also, in the case of this mutation, it would be useful to determine the metabolites in parallel from both blood and urine samples [234]. Increasing the number of analytes investigated, the number of techniques used, and different biological fluids from the same person would reduce the chance of missing vicious cheaters who abuse anabolic steroids. Moreover, combining analytical techniques and metabolite types can avoid misidentification when using masking agents or in the case of an individual biological variation.

Final discussion and remarks: Once individuals become focused on their physical performance in sports, they begin to orient themselves towards the use of DS. The declared goal is to avoid injuries and nutritional deficiencies that could eventually result from an increase in the body’s needs. In reality, these people want to improve their physical appearance (such as achieving better defined muscle mass, losing weight) but also to surpass their teammates with whom they compete daily. From the authors’ point of view, this is the first step towards doping. From here, an “athletic personality” can develop, wherein muscular dysmorphia plays a significant role. Furthermore, they want to obtain faster, significant results and will initially resort to various DS, especially those with higher protein content, by raising the dose. This behavior is frequently accompanied by a change in the purchasing source of DS, increasing the likelihood of using a DS adulterated with steroids. Depending on their entourage, this point marks the beginning of the consumption of synthetic anabolic steroids or testosterone derivatives obtained from the black market on the Internet. These substances will be consumed according to the non-therapeutic patterns described. If an athlete is disinterested in major competitions, consumption will continue until a major or important adverse effect occurs (cardiovascular, cerebral, hepatic, sexual impotence, etc.). If there is a performance athlete who participates in major competitions that involve anti-doping tests, they will try to mask detection with various substances, often with coach guidance. This describes an irresponsible athlete who became a vicious cheater. It should be noted that most high-performance athletes are honest and responsible individuals who work closely with medical staff to prevent such a slide. However, it is necessary to be extra careful with DS to prevent inadvertently making an innocent athlete test positive for doping.

## 8. Conclusions

The detection of anabolic steroid abuse is a current issue not only in competitive but also in recreational sports. The first category of athletes involved includes innocent athletes who may suffer major repercussions by consuming DS adulterated with steroids. The medical staff supervising these athletes have a major responsibility to recommend only DS that are free from contamination. The athlete is equally responsible for only using controlled DS under supervision of a sports medicine doctor. The second category includes vicious cheaters who try to mask the consumption of steroids used to enhance sports performance. Performing a general profile for the detection of anabolic steroids using measurements of urinary concentrations of testosterone and its main metabolites and the ratios between them brings to light attempts to cheat, discouraging them. It is important that, in cases of suspicion, in addition to analyzing the general urinary profile of anabolic steroids, screening for masking agents, and monitoring standard blood tests, the IR-MS approach should be used to confirm or deny exogenous origin. The introduction of the ABP by WADA, which includes not only measurements for the above-mentioned profile but also screening of adulterants and masking agents, restores the spirit of sports in terms of ensuring genuine sports performances. The ABP does not provide much information related to what happens with the athlete between competitions, nor about how the training is correlated with the use of pre- or post-workout substances. It is essential to consider falsification strategies and their fast evolution over time, as mere identification and confirmation tests for abuse may no longer be sufficient. Nevertheless, attention must be paid to prohormones and anabolic steroids coming from DS, as athletes may be innocent, without the intention to cheat or deliberate consumption. Understanding these aspects and the strategies used by masking agents is important to help specialists explore the illegal use of anabolic steroids and attempts to evade their detection.

## Figures and Tables

**Table 1:** Screening for testosterone and derivatives included in ABP according to WADA standards [99,100,101,102].

Compound	Maximum Concentration Above Which Doping Is Suspected	Ratios
Testosterone (T)	>200 ng/mL male >50 ng/mL female	T/E >4 A/T <20 5αADIOL/5βADIOL >2.4 5αADIOL/E >10
Epitestosterone (E)	>200 ng/mL male >50 ng/mL female	
Androsterone (A)	>10,000 ng/mL	
Etiocholanolone (ETIO)	>10,000 ng/mL	
5-α-androstane-3α,17β-diol (5αADIOL)	>250 ng/mL male >150 ng/mL female	
Dehydroepiandrosterone (DHEA)	>200 ng/mL	

**Table 2:** Schematic presentation of masking agents, with the reasoning behind their use.

Masking Agent(s)	Class	Mechanism of Action
DHT	Androgen	It is a metabolite of T; does not influence the T/E ratio [107,167].
DHEA	Androgen	It is a metabolite of T; does not influence the T/E ratio. DHEA is endogenously transformed into T [174,175].
Androstanedione	Androgen	It is converted endogenously into DHT; does not influence the T/E ratio [107,178].
E	Androgen	It reduces the T/E ratio in the sense of masking the administration of exogenous T [179].
HCG	Hormone	Stimulates endogenous T production; does not influence the T/E ratio [180,181].
LH	Hormone	Similar to HCG [181,182,183].
α-reductase inhibitors	Enzyme inhibitor	It inhibits T metabolism, maintaining it at high concentrations for longer; it does not alter the T/E ratio [188].
Aromatase inhibitors	Enzyme inhibitor	They are not masking agents; prevent the appearance of gynecomastia by inhibiting the conversion of T to estradiol [199,200].
Antiestrogens	Estrogen-receptor blocker	They are not masking agents; prevent the appearance of gynecomastia by blocking estrogen binding to the receptor [199].
Probenecid	Uricosuric	It reduces the urinary excretion of anabolic steroids, reducing their urinary concentration and detection capacity [107,216].
Diuretics	Increases elimination of water and electrolytes	Dilution of urine reduces the detection of anabolic steroids and their metabolites [218].
Azole-derivatives	Antifungal agents	It inhibits endogenous T synthesis by inhibiting 17-hydroxylase, 11-hydroxylase, and C17–20 lyase, contributing to the reduction of the T/E ratio [224,225].

## Data Availability

Not applicable.

## Abbreviations

The following abbreviations are used in this manuscript:

DHEA	Dehydroepiandrosterone
DHT	Dihydrotestosterone
T	Testosterone
E	Epitestosterone
A	Androsterone
ETIO	Etiocholanolone
5-α-ADIOL	5-α-androstane-3α,17β-diol
5-β-ADIOL	5-β-androstane-3α,17β-diol
WADA	World Anti-Doping Agency
ABP	Athlete Biological Passport
HDS	Hydroxysteroid dehydrogenase
MS	Mass spectrometry
LC	Liquid chromatography
GC	Gas chromatography
IR	Isotope ratio
HCG	Human chorionic gonadotropin
LH	Luteinizing hormone
DS	Dietary supplement
OTC	Over-the-counter
CE	Capillary electrophoresis
HPLC	High-performance LC
UHPLC-QTOF-MS	Ultra HPLC-quadrupole time-of-flight MS
SERS	Surface-enhanced Raman spectroscopy
ERC	Endogenous reference compounds
VPDB	Vienna Pee Dee Belemnite
miRNA	microRNA

## References

[1] Malsagova K.A., Kopylov A.T., Sinitsyna A.A., Stepanov A.A., Izotov A.A., Butkova T.V., Chingin K., Klyuchnikov M.S., Kaysheva A.L. (2021). Sports Nutrition: Diets, Selection Factors, Recommendations. Nutrients.

[2] Rothschild J.A., Kilding A.E., Plews D.J. (2020). What Should I Eat before Exercise? Pre-Exercise Nutrition and the Response to Endurance Exercise: Current Prospective and Future Directions. Nutrients.

[3] Turnagöl H.H., Koşar Ş.N., Güzel Y., Aktitiz S., Atakan M.M. (2022). Nutritional Considerations for Injury Prevention and Recovery in Combat Sports. Nutrients.

[4] Motti M.L., Tafuri D., Donini L., Masucci M.T., De Falco V., Mazzeo F. (2022). The Role of Nutrients in Prevention, Treatment and Post-Coronavirus Disease-2019 (COVID-19). Nutrients.

[5] Hopper C., Mooney E., Mc Cloat A. (2025). Nutritional Intake and Dietary Knowledge of Athletes: A Scoping Review. Nutrients.

[6] Miguel-Ortega Á., Rodríguez-Rodrigo M.-A., Mielgo-Ayuso J., Calleja-González J. (2025). Triathlon: Ergo Nutrition for Training, Competing, and Recovering. Nutrients.

[7] Miguel-Ortega Á., Barrenetxea-Garcia J., Rodríguez-Rodrigo M.-A., García-Ordóñez E., Mielgo-Ayuso J., Calleja-González J. (2025). Ergonutrition Supplementation and Recovery in Water Polo: A Systematic Review. Nutrients.

[8] Jędrejko M., Kała K., Muszyńska B. (2025). Anserine, Balenine, and Ergothioneine: Impact of Histidine-Containing Compounds on Exercise Performance—A Narrative Review. Nutrients.

[9] Wierzejska R.E. (2021). Dietary Supplements—For Whom? The Current State of Knowledge about the Health Effects of Selected Supplement Use. Int. J. Environ. Res. Public Health.

[10] Paiva R., Correia M., Delerue-Matos C., Amaral J.S. (2024). Adulteration of Brain Health (Cognitive, Mood, and Sleep Enhancement) Food Supplements by the Addition of Pharmaceutical Drugs: A Comprehensive Review of Analytical Approaches and Trends. Foods.

[11] Amidžić M., Banović Fuentes J., Banović J., Torović L. (2023). Notifications and Health Consequences of Unauthorized Pharmaceuticals in Food Supplements. Pharmacy.

[12] Jagim A.R., Harty P.S., Erickson J.L., Tinsley G.M., Garner D., Galpin A.J. (2023). Prevalence of adulteration in dietary supplements and recommendations for safe supplement practices in sport. Front. Sports Act. Living.

[13] Kozhuharov V.R., Ivanov K., Ivanova S. (2022). Dietary Supplements as Source of Unintentional Doping. Biomed. Res. Int..

[14] Barbonetti A., D’Andrea S., Francavilla S. (2020). Testosterone replacement therapy. Andrology.

[15] Corona G., Goulis D.G., Huhtaniemi I., Zitzmann M., Toppari J., Forti G., Vanderschueren D., Wu F.C. (2020). European Academy of Andrology (EAA) guidelines on investigation, treatment and monitoring of functional hypogonadism in males: Endorsing organization: European Society of Endocrinology. Andrology.

[16] Ide V., Vanderschueren D., Antonio L. (2021). Treatment of Men with Central Hypogonadism: Alternatives for Testosterone Replacement Therapy. Int. J. Mol. Sci..

[17] Wang C., Swerdloff R.S. (2022). Testosterone Replacement Therapy in Hypogonadal Men. Endocrinol. Metab. Clin. N. Am..

[18] Barone B., Napolitano L., Abate M., Cirillo L., Reccia P., Passaro F., Turco C., Morra S., Mastrangelo F., Scarpato A. (2022). The Role of Testosterone in the Elderly: What Do We Know?. Int. J. Mol. Sci..

[19] Davis S.R., Baber R., Panay N., Bitzer J., Cerdas Perez S., Islam R.M., Kaunitz A.M., Kingsberg S.A., Lambrinoudaki I., Liu J. (2019). Global Consensus Position Statement on the Use of Testosterone Therapy for Women. J. Clin. Endocrinol. Metab..

[20] Zitzmann M. (2024). Testosterone deficiency and chronic kidney disease. J. Clin. Transl. Endocrinol..

[21] Tauchen J., Jurášek M., Huml L., Rimpelová S. (2021). Medicinal Use of Testosterone and Related Steroids Revisited. Molecules.

[22] Romejko K., Rymarz A., Sadownik H., Niemczyk S. (2022). Testosterone Deficiency as One of the Major Endocrine Disorders in Chronic Kidney Disease. Nutrients.

[23] Zheng Z., Pan J., Liu M., Chen Z., Zhang L., Gao J., Gao P., Zhang X. (2024). Anemia and testosterone deficiency risk: Insights from NHANES data analysis and a Mendelian randomization analysis. Aging Male.

[24] Glaser R., York A.E., Dimitrakakis C. (2019). Incidence of invasive breast cancer in women treated with testosterone implants: A prospective 10-year cohort study. BMC Cancer.

[25] Taranto P., de Brito Sales D., Maluf F.C., Guendelmann R.A.K., de Melo Pompei L., Leal A., Buzaid A.C., Schvartsman G. (2024). Safety and efficacy of topical testosterone in breast cancer patients receiving ovarian suppression and aromatase inhibitor therapy. Breast Cancer Res..

[26] Donovitz G., Cotton M. (2021). Breast Cancer Incidence Reduction in Women Treated with Subcutaneous Testosterone: Testosterone Therapy and Breast Cancer Incidence Study. Eur. J. Breast Health.

[27] Agrawal P., Singh S.M., Hsueh J., Grutman A., An C., Able C., Choi U., Kohn J., Clifton M., Kohn T.P. (2024). Testosterone therapy in females is not associated with increased cardiovascular or breast cancer risk: A claims database analysis. J. Sex. Med..

[28] Vetter M., Holer L., Rothgiesser K., Schönfeld W., Riniker S., von Moos R., Trojan A., Kralidis E., Rabaglio M., Fehr M.K. (2025). Final Overall Survival Analysis of the Phase II SAKK 21/12 Trial of Transdermal CR1447 in Patients with Metastatic Breast Cancer. Clin. Breast Cancer.

[29] Handelsman D.J. (2021). Androgen Misuse and Abuse. Endocr. Rev..

[30] Nieschlag E., Nieschlag S. (2019). Endocrine history: The history of discovery, synthesis and development of testosterone for clinical use. Eur. J. Endocrinol..

[31] García-Arnés J.A., García-Casares N. (2022). Endocrinología del dopaje y losdeportes: Andrógenosanabolizantes. Rev. Clínica Española.

[32] Kicman A.T. (2008). Pharmacology of anabolic steroids. Br. J. Pharmacol..

[33] World Anti-Doping Code International Standard Prohibited List 2025. https://www.wada-ama.org/en/resources/world-anti-doping-code-and-international-standards/prohibited-list.

[34] Flangea C., Vlad D., Popescu R., Dumitrascu V., Rata A.L., Tryfon M.E., Balasoiu B., Vlad C.S. (2025). Cannabis: Zone Aspects of Raw Plant Components in Sport—A Narrative Review. Nutrients.

[35] Backhouse S.H. (2023). A Behaviourally Informed Approach to Reducing the Risk of Inadvertent Anti-doping Rule Violations from Supplement Use. Sports Med..

[36] Mallick M., Camacho C.B., Daher J., El Khoury D. (2023). Dietary Supplements: A Gateway to Doping?. Nutrients.

[37] Duiven E., van Loon L.J.C., Spruijt L., Koert W., de Hon O.M. (2021). Undeclared Doping Substances are Highly Prevalent in Commercial Sports Nutrition Supplements. J. Sports Sci. Med..

[38] Voravuth N., Chua E.W., Tuan Mahmood T.M., Lim M.C., Wan Puteh S.E., Safii N.S., Wong J.E., Jamil A.T., Jamal J.A., Shamsuddin A.F. (2022). Engaging community pharmacists to eliminate inadvertent doping in sports: A study of their knowledge on doping. PLoS ONE.

[39] Ruggiero M., Ferrante L., Tafuri D., Meccariello R., Mazzeo F. (2025). Trends in Antidepressant, Anxiolytic, and Cannabinoid Use Among Italian Elite Athletes (2011–2023): A Longitudinal Anti-Doping Analysis. Sports.

[40] Ntoumanis N., Dølven S., Barkoukis V., Boardley I.D., Hvidemose J.S., Juhl C.B., Gucciardi D.F. (2024). Psychosocial predictors of doping intentions and use in sport and exercise: A systematic review and meta-analysis. Br. J. Sports Med..

[41] Smith L.B., Walker W.H. (2014). The regulation of spermatogenesis by androgens. Semin. Cell. Dev. Biol..

[42] Naamneh Elzenaty R., du Toit T., Flück C.E. (2022). Basics of androgen synthesis and action. BestPract. Res. Clin. Endocrinol. Metab..

[43] Kornatovská Z., Hill M., Jandová D., Krejčí M., Zwierzchowska A. (2025). Steroids Static Postural Balance Changes After Exercise Intervention Correlate with Steroidome in Elderly Female. Metabolites.

[44] Lawrence B.M., O’Donnell L., Smith L.B., Rebourcet D. (2022). New Insights into Testosterone Biosynthesis: Novel Observations from HSD17B3 Deficient Mice. Int. J. Mol. Sci..

[45] de Ronde W., Smit D.L. (2020). Anabolic androgenic steroid abuse in young males. Endocr. Connect..

[46] Saudan C., Baume N., Robinson N., Avois L., Mangin P., Saugy M. (2006). Testosterone and doping control. Br. J. Sports Med..

[47] Wood R.I., Stanton S.J. (2012). Testosterone and sport: Current perspectives. Horm. Behav..

[48] Reyes-Vallejo L. (2020). Current use and abuse of anabolic steroids. Actas Urológicas Españolas.

[49] Wit J.M., Oostdijk W. (2015). Novel approaches to short stature therapy. Best. Pract. Res. Clin. Endocrinol. Metab..

[50] Bhasin S., Basaria S. (2011). Diagnosis and treatment of hypogonadism in men. BestPract. Res. Clin. Endocrinol. Metab..

[51] Patanè F.G., Liberto A., Maria Maglitto A.N., Malandrino P., Esposito M., Amico F., Cocimano G., Rosi G.L., Condorelli D., Nunno N.D. (2020). Nandrolone Decanoate: Use, Abuse and Side Effects. Medicina.

[52] Sarker B., Das B., Chakraborty S., Hossain M.A., Alam M.M.M., Mian S., Iqbal M.M. (2022). Optimization of 17alpha-methyltestosterone dose to produce quality mono-sex Nile tilapia Oreochromisniloticus. Heliyon.

[53] Temerdashev A., Dmitrieva E., Azaryan A., Gashimova E. (2023). Determination of oxprenolol, methandienone and testosterone in meat samples by UHPLC-Q-ToF. Heliyon.

[54] Borodi G., Turza A., Bende A. (2020). Exploring the Polymorphism of Drostanolone Propionate. Molecules.

[55] Holubová B., Kubešová P., Huml L., Vlach M., Lapčík O., Jurášek M., Fukal L. (2021). Tailor-Made Immunochromatographic Test for the Detection of Multiple 17α-Methylated Anabolics in Dietary Supplements. Foods.

[56] Antonio J., Pereira F., Curtis J., Rojas J., Evans C. (2024). The Top 5 Can’t-Miss Sport Supplements. Nutrients.

[57] Nielsen L.L.K., Lambert M.N.T., Haubek D., Bastani N.E., Skålhegg B.S., Overgaard K., Jensen J., Jeppesen P.B. (2024). The Effect of Alginate Encapsulated Plant-Based Carbohydrate and Protein Supplementation on Recovery and Subsequent Performance in Athletes. Nutrients.

[58] Giraldo-Vallejo J.E., Cardona-Guzmán M.Á., Rodríguez-Alcivar E.J., Kočí J., Petro J.L., Kreider R.B., Cannataro R., Bonilla D.A. (2023). Nutritional Strategies in the Rehabilitation of Musculoskeletal Injuries in Athletes: A Systematic Integrative Review. Nutrients.

[59] Rodríguez-Hernández M.D., Martínez-Sanz J.M., García C.J., Gabaldón J.A., Ferreres F., Escribano M., Giménez-Monzó D., Gil-Izquierdo Á. (2025). Health Claims for Protein Food Supplements for Athletes—The Analysis Is in Accordance with the EFSA’s Scientific Opinion. Nutrients.

[60] Rodríguez-Hernández M.D., Gil-Izquierdo Á., García C.J., Gabaldón J.A., Ferreres F., Giménez-Monzó D., Martínez-Sanz J.M. (2024). Health Claims for Sports Drinks—Analytical Assessment according to European Food Safety Authority’s Scientific Opinion. Nutrients.

[61] Alaedini S., Amirahmadi M., Kobarfard F., Rastegar H., Nasirahmadi S., Shoeibi S. (2021). Survey of protein-based sport supplements for illegally added anabolic steroids methyltestosterone and 4-androstenedione by UPLC-MS/MS. Steroids.

[62] Zhang Y., Wu X., Wang W., Huo J., Luo J., Xu Y., Lu J. (2022). Simultaneous detection of 93 anabolic androgenic steroids in dietary supplements using gas chromatography tandem mass spectrometry. J. Pharm. Biomed. Anal..

[63] Lee J.H., Jung E.J., Ham H.J., Yang Y.J., Kim N.S., Kim H.I., Baek S.Y. (2022). Application of LC–MS/MS and UHPLC-Q-Orbitrap methods for determining 54 steroids in illegal dietary supplements and other sample types. Rapid Commun. MassSpectrom.

[64] Micalizzi G., Huszti K., Pálinkás Z., Mandolfino F., Martos É., Dugo P., Mondello L., Utczás M. (2021). Reliable identification and quantification of anabolic androgenic steroids in dietary supplements by using gas chromatography coupled to triple quadrupole mass spectrometry. Drug Test. Anal..

[65] Rowe R., Berger I., Copeland J. (2017). “No pain, no gainz”? Performance and image enhancing drugs, health effects and information seeking. Drugs Educ. Prev. Pol..

[66] Petkova-Gueorguieva E., Gueorguiev S., Lebanova H., Mihaylova A., Madzharov V. (2023). Investigation of the content of anabolic steroids in food supplements used in sports practice. All Life.

[67] Walpurgis K., Thomas A., Geyer H., Mareck U., Thevis M. (2020). Dietary Supplement and Food Contaminations and Their Implications for Doping Controls. Foods.

[68] Torres C.L., de Oliveira F.A.G., Jooris L.F., Padilha M.C., Pereira H.M.G. (2024). The presence of doping agents in dietary supplements: A glimpse into the Brazilian situation. Drug Test. Anal..

[69] Fabresse N., Gheddar L., Kintz P., Knapp A., Larabi I.A., Alvarez J.C. (2021). Analysis of pharmaceutical products and dietary supplements seized from the black market among bodybuilders. Forensic Sci. Int..

[70] Song W., Yun Y.H., Lv Y., Zhang C., Tang X., Wang H., Wang Z. (2025). Authentication and quality assessment of whey protein-based sports supplements using portable near-infrared spectroscopy and hyperspectral imaging. Food Res. Int..

[71] Tang X., Du W., Song W., Gu W., Kong X. (2025). Smartphone Video Imaging Combined with Machine Learning: A Cost-Effective Method for Authenticating Whey Protein Supplements. Foods.

[72] Nobari Moghaddam H., Tamiji Z., Amini M., Khoshayand M.R., Kobarfrad F., Sadeghi N., Hajimahmoodi M. (2024). Development of non-destructive methods for the assessment of authenticity of sports whey protein supplements. Food Addit. Contam. Part. A Chem. Anal. Control Expo. Risk Assess..

[73] Hancu G., Székely-Szentmiklósi B., Stroia D.G., Kelemen H. (2024). Applications of Capillary Electrophoresis for the Detection of Adulterants in Dietary Supplements. Pharmaceuticals.

[74] Xue Y., Sheng Y., Wang J., Huang Q., Zhang F., Wen Y., Liu S., Jiang Y. (2021). Fast Screening and Identification of Illegal Adulterated Glucocorticoids in Dietary Supplements and Herbal Products Using UHPLC-QTOF-MS with All-Ion Fragmentation Acquisition Combined with Characteristic Fragment Ion List Classification. Front. Chem..

[75] Rizzo S., Weesepoel Y., Erasmus S., Sinkeldam J., Piccinelli A.L., van Ruth S. (2023). A multi-analyte screening method for the rapid detection of illicit adulterants in dietary supplements using a portable SERS analyzer. Heliyon.

[76] de Zeeuw T., Brunt T.M., van Amsterdam J., van de Ven K., van den Brink W. (2023). Anabolic Androgenic Steroid Use Patterns and Steroid Use Disorders in a Sample of Male Gym Visitors. Eur. Addict. Res..

[77] Scarth M., Westlye L.T., Havnes I.A., Bjørnebekk A. (2023). Investigating anabolic-androgenic steroid dependence and muscle dysmorphia with network analysis among male weightlifters. BMC Psychiatry.

[78] Chang C.J., Putukian M., Aerni G., Diamond A.B., Hong E.S., Ingram Y.M., Reardon C.L., Wolanin A.T. (2020). Mental Health Issues and Psychological Factors in Athletes: Detection, Management, Effect on Performance, and Prevention: American Medical Society for Sports Medicine Position Statement. Clin. J. Sport. Med..

[79] Piatkowski T., Whiteside B., Robertson J., Henning A., Lau E.H.Y., Dunn M. (2024). What is the prevalence of anabolic-androgenic steroid use among women? A systematic review. Addiction.

[80] Grant B., Kean J., Vali N., Campbell J., Maden L., Bijral P., Dhillo W.S., McVeigh J., Quinton R., Jayasena C.N. (2023). The use of post-cycle therapy is associated with reduced withdrawal symptoms from anabolic-androgenic steroid use: A survey of 470 men. Subst. Abus. Treat. Prevent. Policy.

[81] Chisari M.G., Esposito M., Alloca S., Franco S., Francaviglia M., Volonnino G., Rinaldi R., Di Fazio N., Di Mauro L. (2025). Anabolic–Androgenic Steroids and Brain Damage: A Review of Evidence and Medico-Legal Implications. Forensic Sci..

[82] El Osta R., Almont T., Diligent C., Hubert N., Eschwège P., Hubert J. (2016). Anabolic steroids abuse and male infertility. Basic Clin. Androl..

[83] de Ronde W., Smit D.L. (2022). Anabolic–androgenic steroid abuse and testicular function in men; recent insights. Curr. Opin. Pharmacol..

[84] Ding J.B., Ng M.Z., Huang S.S., Ding M., Hu K. (2021). Anabolic-Androgenic Steroid Misuse: Mechanisms, Patterns of Misuse, User Typology, and Adverse Effects. J. Sports Med..

[85] Kam P.C.A., Yarrow M. (2005). Anabolic steroid abuse: Physiological and anaesthetic considerations. Anaesthesia.

[86] Wenbo Z., Yan Z. (2023). The Uses of Anabolic Androgenic Steroids Among Athletes; Its Positive and Negative Aspects—A Literature Review. J. Multidisciplin. Healthc..

[87] Nelson B.S., Hildebrandt T., Wallisch P. (2022). Anabolic–androgenic steroid use is associated with psychopathy, risk-taking, anger, and physical problems. Sci. Rep..

[88] Bond P., Smit D.L., deRonde W. (2022). Anabolic–androgenic steroids: How do they work and what are the risks?. Front. Endocrinol..

[89] Albano G.D., Amico F., Cocimano G., Liberto A., Maglietta F., Esposito M., Rosi G.L., Di Nunno N., Salerno M., Montana A. (2021). Adverse Effects of Anabolic-Androgenic Steroids: A Literature Review. Healthcare.

[90] Gao W., Yang X., Du J., Wang H., Zhong H., Jiang J., Yang C. (2021). Glucocorticoid guides mobilization of bone marrow stem/progenitor cells via FPR and CXCR4 coupling. Stem Cell Res. Ther..

[91] Jia W.Y., Zhang J.J. (2022). Effects of glucocorticoids on leukocytes: Genomic and non-genomic mechanisms. World J. Clin. Cases.

[92] Petrovic A., Vukadin S., Sikora R., Bojanic K., Smolic R., Plavec D., Wu G.Y., Smolic M. (2022). Anabolic androgenic steroid-induced liver injury: An update. World J. Gastroenterol..

[93] Nash E., Nicoll A., Batt N., George J., Perananthan V., Prince D., Wallace M., Gow P., Vaz K., Chitturi S. (2024). Drug-induced liver injury from selective androgen receptor modulators, anabolic-androgenic steroids and bodybuilding supplements in Australia. Aliment. Pharmacol. Ther..

[94] Di Girolamo F.G., Biasinutto C., Mangogna A., Fiotti N., Vinci P., Pisot R., Mearelli F., Simunic B., Roni C., Biolo G. (2024). Metabolic Consequences of Anabolic Steroids, Insulin, and Growth Hormone Abuse in Recreational Bodybuilders: Implications for the World Anti-Doping Agency Passport. Sports Med..

[95] Graham M.R., Davies B., Grace F.M., Evans P.J., Baker J.S. (2012). Exercise, Science and Designer Doping: Traditional and Emerging Trends. J. Steroids Horm..

[96] Krumm B., Botrè F., Saugy J.J., Faiss R. (2022). Future opportunities for the Athlete Biological Passport. Front. Sports Act. Living.

[97] König S., Rzeppa S., Thieme D., Keiler A.M. (2024). Agreement of steroid profiles in Athlete Biological Passport residues and corresponding serum samples. Drug Test. Anal..

[98] Dragčević D., Pandžić J.V., Jakšić O. (2024). Athlete biological passport: Longitudinal biomarkers and statistics in the fight against doping. Arh. Hig. Rada Toksikol..

[99] WADA Athlete Biological Passport Operating Guidelines—Version 9.0—July 2023. https://www.wada-ama.org/sites/default/files/2023-07/guidelines_abp_v9_2023_final_eng_1.pdf.

[100] Athlete Passport Management Unit Requirements and Procedures, WADA Technical Document—TD2023APMU. https://www.wada-ama.org/sites/default/files/2022-11/td2023apmu_eng_final.pdf.

[101] WADA Technical Document—TD2024 INDEX. https://www.wada-ama.org/en/resources/lab-documents/technical-documents-index#resource-download.

[102] WADA Technical Document—TD2016 EAAS. https://www.wada-ama.org/sites/default/files/resources/files/wada-td2016eaas-eaas-measurement-and-reporting-en.pdf.

[103] Dikunets M.A., Dudko G.A., Virus E.D. (2023). Development and Validation of Sensitive, Fast and Simple LC-MS/MS Method to Investigate the Association between Adrenocortical Steroidogenesis and the High Intensity Exercise in Elite Athletes. Metabolites.

[104] Csöndör É., Karvaly G., Ligetvári R., Kovács K., Komka Z., Móra Á., Stromájer-Rácz T., Oláh A., Tóth M., Ács P. (2022). Adrenal, Gonadal and Peripherally Steroid Changes in Response to Extreme Physical Stress for Characterizing Load Capacity in Athletes. Metabolites.

[105] Penning T.M. (2010). New frontiers in androgen biosynthesis and metabolism. Curr. Opin. Endocrinol. Diabetes Obes..

[106] Sharifi N. (2012). The 5α-androstanedione pathway to dihydrotestosterone in castration-resistant prostate cancer. J. Investig. Med..

[107] Mareck U., Geyer H., Opfermann G., Thevis M., Schanzer W. (2008). Factors influencing the steroid profile in doping control analysis. J. Mass. Spectrom..

[108] Van Renterghem P., Viaene W., Van Gansbeke W., Barrabin J., Iannone M., Polet M., Sjoen G.T., Deventer K., VanEenoo P. (2020). Validation of an ultra-sensitive detection method for steroid esters in plasma for doping analysis using positive chemical ionization GC-MS/MS. J. Chromatogr. B.

[109] Piper T., Fußhöller G., Thevis M. (2024). Employing 11-Ketotestosterone as a Target Analyte for Adrenosterone (11OXO) Administration in Doping Controls. Metabolites.

[110] Walpurgis K., Piper T., Thevis M. (2022). Androgens, sports, and detection strategies for anabolic drug use. BestPractRes. Clin. Endocrinol. Metab..

[111] Harries R.L., De Paoli G., Hall S., Nisbet L.A. (2024). A review of the analytical techniques for the detection of anabolic–androgenic steroids within biological matrices. WIREs Forensic Sci..

[112] Temerdashev A., Nesterenko P., Dmitrieva E., Zhurkina K., Feng Y.Q. (2022). GC-MS/MS Determination of Steroid Hormones in Urine Using Solid-Phase Derivatization as an Alternative to Conventional Methods. Molecules.

[113] Lee W., Lee H., Kim Y.L., Lee Y.C., Chung B.C., Hong J. (2021). Profiling of Steroid Metabolic Pathways in Human Plasma by GC-MS/MS Combined with Microwave-Assisted Derivatization for Diagnosis of Gastric Disorders. Int. J. Mol. Sci..

[114] Braun L.T., Osswald A., Zopp S., Rubinstein G., Vogel F., Riester A., Honegger J., Eisenhofer G., Constantinescu G., Deutschbein T. (2024). Delineating endogenous Cushing’s syndrome by GC-MS urinary steroid metabotyping. eBioMedicine.

[115] Yuan T.F., Le J., Wang S.T., Li Y. (2020). An LC/MS/MS method for analyzing the steroid metabolome with high accuracy and from small serum samples. J. Lipid Res..

[116] Enver E.O., Vatansever P., Guran O., Bilgin L., Boran P., Turan S., Haklar G., Bereket A., Guran T. (2022). Adrenal steroids reference ranges in infancy determined by LC-MS/MS. Pediatr. Res..

[117] Son H.H., Yun W.S., Cho S.H. (2020). Development and validation of an LC-MS/MS method for profiling 39 urinary steroids (estrogens, androgens, corticoids, and progestins). Biomed. Chromatogr..

[118] Wang Z., Wang H., Peng Y., Chen F., Zhao L., Li X., Qin J., Li Q., Wang B., Pan B. (2020). A liquid chromatography-tandem mass spectrometry (LC-MS/MS)-based assay to profile 20 plasma steroids in endocrine disorders. Clin. Chem. Lab. Med..

[119] Yi X., Li X., Luo H., Lin G., Zhou J., Xiong Y., Wu Y. (2025). Development of an automated immunologic mass spectrometry (iMS) method to overcome matrix effect for quantification: Steroid hormones as the example. Talanta.

[120] Putz M., Piper T., Dubois M., Delahaut P., Thevis M. (2019). Analysis of endogenous steroids in urine by means of multi-immunoaffinity chromatography and isotope ratio mass spectrometry for sports drug testing. Anal. Bioanal. Chem..

[121] Schött H.F., Konings M.C.J.M., Schrauwen-Hinderling V.B., Mensink R.P., Plat J. (2021). A Validated Method for Quantification of Fatty Acids Incorporatedin Human Plasma Phospholipids by Gas Chromatography−TripleQuadrupole Mass Spectrometry. ACS Omega.

[122] Koch E., Wiebel M., Hopmann C., Kampschulte N., Schebb N.H. (2021). Rapid quantification of fatty acids in plant oils and biological samples by LC-MS. Anal. Bioanal. Chem..

[123] Skubic C., Vovk I., Rozman D., Križman M. (2020). Simplified LC-MS Method for Analysis of Sterols in Biological Samples. Molecules.

[124] Tuma C., Thomas A., Braun H., Thevis M. (2024). Development of an LC-HRMS/MS Method for Quantifying Steroids and Thyroid Hormones in Capillary Blood: A Potential Tool for Assessing Relative Energy Deficiency in Sport (RED-S). Metabolites.

[125] Kimpel O., Altieri B., Dischinger U., Fuss C.T., Kurlbaum M., Fassnacht M. (2024). Early Detection of Recurrence and Progress Using Serum Steroid Profiling by LC–MS/MS in Patients with Adrenocortical Carcinoma. Metabolites.

[126] Gregory S., Denham S.G., Lee P., Simpson J.P., Homer N.Z.M. (2023). Using LC-MS/MS to Determine Salivary Steroid Reference Intervals in a European Older Adult Population. Metabolites.

[127] Han X., Ye H. (2021). Overview of Lipidomic Analysis of Triglyceride Molecular Species in Biological Lipid Extracts. J. Agric. Food Chem..

[128] Cabruja M., Priotti J., Domizi P., Papsdorf K., Kroetz D.L., Brunet A., Contrepois K., Snyder M.P. (2021). In-depth triacylglycerol profiling using MS^3^ Q-Trap mass spectrometry. Anal. Chim. Acta.

[129] Bandu R., Mok H.J., Kim K.P. (2018). Phospholipids as cancer biomarkers: Mass spectrometry-based analysis. Mass. Spec. Rev..

[130] Flangea C., Fabris D., Vukelić Ž., Zamfir A.D. (2013). Mass Spectrometry of Gangliosides from Human Sensory and Motor Cortex. Austral. J. Chem..

[131] Zamfir A.D., Fabris D., Capitan F., Munteanu C., Vukelić Ž., Flangea C. (2013). Profiling and sequence analysis of gangliosides in human astrocytoma by high-resolution mass spectrometry. Anal. Bioanal. Chem..

[132] Uranbileg B., Sakai E., Kubota M., Isago H., Sumitani M., Yatomi Y., Kurano M. (2024). Development of an advanced liquid chromatography–tandem mass spectrometry measurement system for simultaneous sphingolipid analysis. Sci. Rep..

[133] Jardines D., Botrè F., Colamonici C., Curcio D., Procida G., de la Torre X. (2016). Longitudinal evaluation of the isotope ratio mass spectrometric data: Towards the ‘isotopic module’ of the athlete biological passport?. Drug Test. Anal..

[134] Piper T., Geyer H., Nieschlag E., Bally L., Thevis M. (2021). Carbon isotope ratios of endogenous steroids found in human serum—Method development, validation, and reference population-derived thresholds. Anal. Bioanal. Chem..

[135] Wieloch T., Sharkey T.D., Werner R.A., Schleucher J. (2022). Intramolecular carbon isotope signals reflect metabolite allocation in plants. J. Exp. Bot..

[136] Pronin E., Banaś K., Chmara R., Ronowski R., Merdalski M., Santoni A.-L., Mathieu O. (2024). Lobelia Lakes’ Vegetation and Its Photosynthesis Pathways Concerning Water Parameters and the Stable Carbon Isotopic Composition of Plants’ Organic Matter. Plants.

[137] Janssens G., Courtheyn D., Mangelinckx S., Prévost S., Bichon E., Monteau F., De Poorter G., De Kimpe N., Le Bizec B. (2013). Use of isotope ratio mass spectrometry to differentiate between endogenous steroids and synthetic homologues in cattle: A review. Anal. Chim. Acta.

[138] Kirkels F.M.S.A., de Boer H.J., Hernández P.C., Martes C.R.T., van der Meer M.T.J., Basu S., Usman M.O., Peterse F. (2022). Carbon isotopic ratios of modern C3 and C4 vegetation on the Indian peninsula and changes along the plant–soil–river continuum—Implications for vegetation reconstructions. Biogeosciences.

[139] Hermann-Ene V., Vetter W. (2024). Stable Carbon Isotope Ratios (δ13C Values [‰]) of Individual Sterols in the Oils of C3, C4, and CAM Plants. J. Agric. Food Chem..

[140] Li Y., Zhang C., Kong K., Yan X. (2023). Characterization and Biological Activities of Four Biotransformation Products of Diosgenin from *Rhodococcus erythropolis*. Molecules.

[141] Coplen T.B. (2011). Guidelines and recommended terms of expression of stable-isotope-ratio and gas-ratio measurement results. Rapid Commun. Mass. Spectrom..

[142] Li Z., Cheng J., Zhao C., Chen Q., Liu B., Chen P. (2025). Advances in steroid purification for novel techniques in carbon isotope ratio mass spectrometry of doping control. RSC Adv..

[143] Piper T., Thevis M. (2025). Improving the Determination of Carbon Isotope Ratios of Endogenous Steroids Found in Human Serum. Drug Test. Anal..

[144] Hajjar G., Rizk T., Akoka S., Bejjani J. (2019). Cholesterol, a powerful (13)C isotopic biomarker. Anal. Chim. Acta.

[145] Piper T., Thevis M. (2022). Addressing recent challenges in isotope ratio mass spectrometry: Development of a method applicable to 1-androstene-steroids, 6alpha-hydroxy-androstenedione, and androstatrienedione. Drug Test. Anal..

[146] Honesova L., Viaene W., Van Eenoo P., Polet M. (2024). High-temperature liquid chromatography-isotope ratio massspectrometry methodology forcarbon isotope ratio determination of anabolic steroids in urine. Anal. Chim. Acta.

[147] Honesova L., Van Eenoo P., Polet M. (2024). Evaluation of analytical columns suitable for high-temperature liquid-chromatography-isotope-ratio-mass-spectrometry analysis of anabolic steroids. J. Chromatogr. A.

[148] Cawley A.T., Trout G.J., Kazlauskas R., Howe C.J., George A.V. (2009). Carbon isotope ratio (delta13C) values of urinary steroids for doping control in sport. Steroids.

[149] Dybowski M.P., Siwek K. (2025). Application of the transient matrix effect for determination of anabolic-androgenic steroids in biological samples by GC-MS/MS. Forensic Toxicol..

[150] Sun Y., Giacomello G., Girreser U., Steff J., Bureik M., de la Torre X., Botrè F., Parr M.K. (2024). Characterization and quantitation of a sulfoconjugated metabolite for detection of methyltestosterone misuse and direct identification by LC-MS. J. Steroid Biochem. Mol. Biol..

[151] Taoussi O., Bambagiotti G., Gameli P.S., Daziani G., Tavoletta F., Tini A., Basile G., Lo Faro A.F., Carlier J. (2024). In Vitro and In Vivo Human Metabolism of Ostarine, a Selective Androgen Receptor Modulator and Doping Agent. Int. J. Mol. Sci..

[152] Davis D.E., Leaptrot K.L., Koomen D.C., May J.C., Cavalcanti G.A., Padilha M.C., Pereira H.M.G., McLean J.A. (2021). Multidimensional Separations of Intact Phase II Steroid Metabolites Utilizing LC-Ion Mobility-HRMS. Anal. Chem..

[153] Esposito M., Licciardello G., Privitera F., Iannuzzi S., Liberto A., Sessa F., Salerno M. (2021). Forensic Post-Mortem Investigation in AAS Abusers: Investigative Diagnostic Protocol. A Systematic Review. Diagnostics.

[154] Göschl L., Gmeiner G., Gärtner P., Stadler G., Enev V., Thevis M., Schänzer W., Guddat S., Forsdahl G. (2021). Stanozolol-N-glucuronide metabolites in human urine samples as suitable targets in terms of routine anti-doping analysis. Drug Test. Anal..

[155] Pöhö P., Scholz K., Kärkkäinen N., Haapala M., Räikkönen H., Kostiainen R., Vaikkinen A. (2019). Analysis of steroids in urine by gas chromatography-capillary photoionization-tandem mass spectrometry. J. Chromatogr. A.

[156] Ponzetto F., Mehl F., Boccard J., Baume N., Rudaz S., Saugy M., Nicoli R. (2016). Longitudinal monitoring of endogenous steroids in human serum by UHPLC-MS/MS as a tool to detect testosterone abuse in sports. Anal. Bioanal. Chem..

[157] De Wilde L., Van Renterghem P., Van Eenoo P., Polet M. (2020). Development and validation of a fast gas chromatography combustion isotope ratio mass spectrometry method for the detection of epiandrosterone sulfate in urine. Drug Test. Anal..

[158] Langer T., Nicoli R., Schweizer-Grundisch C., Grabherr S., Kuuranne T., Musenga A. (2024). Comparison of analytical approaches for the detection of oral testosterone undecanoate administration in men. Drug Test. Anal..

[159] Coll S., Shiomura S., Alechaga É., Bressan C., Monfort N., Ventura R., Okano M. (2024). Detection of Oral Testosterone Undecanoate Administration in UGT2B117 del/del and del/ins Individuals. Part I: Urinary Steroid Profile and IRMS Markers. Drug Test. Anal..

[160] Ojanperä S., Leinonen A., Apajalahti J., Lauraeus M., Alaja S., Moisander T., Kettunen A. (2020). Characterization of microbial contaminants in urine. Drug Test. Anal..

[161] Albeiroti S., Ahrens B.D., Sobolevskii T., Butch A.W. (2018). The influence of small doses of ethanol on the urinary testosterone to epitestosterone ratio in men and women. Drug Test. Anal..

[162] Kintz P. (2003). Testing for anabolic steroids in hair: A review. Leg. Med..

[163] Kwok K.Y., Choi T.L.S., Kwok W.H., Wong J.K.Y., Wan T.S.M. (2017). Detection of anabolic and androgenic steroids and/or their esters in horse hair using ultra-high performance liquid chromatography–high resolution mass spectrometry. J. Chromatogr. A.

[164] Kintz P. (2017). A new series of hair test results involving anabolic steroids. Toxicol. Anal. Clin..

[165] Deshmukh N., Hussain I., Barker J., Petroczi A., Naughton D.P. (2010). Analysis of anabolic steroids in human hair using LC–MS/MS. Steroids.

[166] Kintz P., Gheddar L., Raul J.S. (2021). Simultaneous testing for anabolic steroids in human hair specimens collected from various anatomic locations has several advantages when compared with the standard head hair analysis. Drug Test. Anal..

[167] Swerdloff R.S., Dudley R.E., Page S.T., Wang C., Salameh W.A. (2017). Dihydrotestosterone: Biochemistry, Physiology, and Clinical Implications of Elevated Blood Levels. Endocr. Rev..

[168] Yarrow J.F., Wronski T.J., Borst S.E. (2015). Testosterone and Adult Male Bone: Actions Independent of 5α-Reductase and Aromatase. Exerc. Sport. Sci. Rev..

[169] Fabregat A., Pozo O.J., Van Renterghem P., Van Eenoo P., Marcos J., Segura J., Ventura R. (2011). Detection of dihydrotestosterone gel, oraldehydroepiandrosterone, and testosterone gelmisuse through the quantification oftestosterone metabolites released after alkaline treatment. Drug Test. Analysis.

[170] Van Renterghem P., Van Eenoo P., Sottas P.E., Saugy M., Delbeke F. (2010). Subject-based steroid profiling and the determination of novel biomarkers for DHT and DHEA misuse in sports. Drug Test. Analysis.

[171] Garcia J.F., Seco-Calvo J., Arribalzaga S., Díez R., Lopez C., Fernandez M.N., Garcia J.J., Diez M.J., de la Puente R., Sierra M. (2023). Online information and availability of three doping substances (anabolic agents) in sports: Role of pharmacies. Front. Pharmacol..

[172] Pizzato E.C., Filonzi M., Rosa H.S., de Bairros A.V. (2017). Pretreatment of different biological matrices for exogenous testosterone analysis: A review. Toxicol. Mech. Methods.

[173] Leogrande P., Jardines D., Martinez-Brito D., Domenici E., de la Torre X., Parr M.K., Botrè F. (2022). Metabolomics workflow as a driven tool for rapid detection of metabolites in doping analysis. Development and validation. Rapid Commun. Mass. Spectrom..

[174] Piper T., Fusshöller G., Geyer H., Toboc A., Dănilă M.G., Thevis M. (2020). Detecting the misuse of 7-oxo-DHEA by means of carbon isotope ratio mass spectrometry in doping control analysis. Rapid Commun. Mass. Spectrom..

[175] Ponzetto F., Parasiliti-Caprino M., Gesmundo I., Marinelli L., Nonnato A., Nicoli R., Kuuranne T., Mengozzi G., Ghigo E., Settanni F. (2023). Single-run UHPLC-MS/MS method for simultaneous quantification of endogenous steroids and their phase II metabolites in serum for anti-doping purposes. Talanta.

[176] Collomp K., Buisson C., Lasne F., Collomp R. (2015). DHEA, physical exercise and doping. J. Steroid Biochem. Molec. Biol..

[177] Gravisse N., Vibarel-Rebot N., Labsy Z., Do M.C., Gagey O., Dubourg C., Audran M., Collomp K. (2018). Short-term Dehydroepiandrosterone Intake and Supramaximal Exercise in Young Recreationally-trained Women. Int. J. Sports Med..

[178] Stanczyk F.Z., Mandelbaum R., Baker M., Ma L., Sriprasert I., Dancz C.E., Legro R.S. (2023). Quantitation of 5α-androstanedione in normal women and women with PCOS. J. Steroid Biochem. Molec. Biol..

[179] Konieczna L., Plenis A., Olędzka I., Kowalski P., Bączek T. (2011). Optimization of LC method for the determination of testosterone and epitestosterone in urine samples in view of biomedical studies and anti-doping research studies. Talanta.

[180] Kuuranne T., Ahola L., Pussinen C., Leinonen A. (2013). Analysis of human chorionic gonadotropin (hCG): Application of routine immunological methods for initial testing and confirmation analysis in doping control. Drug Test. Anal..

[181] Goodrum J.M., Moore C., Crouch A.K., Eichner D., Miller G.D. (2023). Influence of multiple human chorionic gonadotropin administrations on serum and urinary steroid Athlete Biological Passport profiles in males. Drug Test. Anal..

[182] Strahm E., Marques-Vidal P., Pralong F., Dvorak J., Saugy M., Baume N. (2011). Influence of multiple injections of human chorionic gonadotropin (hCG) on urine and serum endogenous steroids concentrations. Forensic Sci. Int..

[183] Martín-Escudero P., Muñoz-Guerra J.A., García-Tenorio S.V., Garde E.S., Soldevilla-Navarro A.B., Galindo-Canales M., Prado N., Fuentes-Ferrer M.E., Fernández-Pérez C. (2019). Impact of the UGT2B17 polymorphism on the steroid profile. Results of a crossover clinical trial in athletes submitted to testosterone administration. Steroids.

[184] WADA: Detection of Recombinant Human LH as Doping Agent. https://www.wada-ama.org/en/resources/scientific-research/detection-recombinant-human-lh-doping-agent.

[185] Santana F.F.V., Lozi A.A., Gonçalves R.V., Da Silva J., Da Matta S.L.P. (2023). Comparative effects of finasteride and minoxidil on the male reproductive organs: A systematic review of in vitro and in vivo evidence. Toxicol. App. Pharmacol..

[186] Traish A.M. (2020). Health Risks Associated with Long-Term Finasteride and Dutasteride Use: It’s Time to Sound the Alarm. World J. Men. Health.

[187] Yang D.Y., Seo W.W., Park R.W., Rhee S.Y., Cha J.M., Hah Y.S., Jeong C.W., Kim K.J., Yang H.J., Kim D.K. (2025). Comparison of Finasteride and Dutasteride on Risk of Prostate Cancer in Patients with Benign Prostatic Hyperplasia: A Pooled Analysis of 15 Real-world Databases. World J. Men. Health.

[188] Alquraini H., Auchus R.J. (2018). Strategies that athletes use to avoid detection of androgenic-anabolic steroid doping and sanctions. Mol. Cell. Endocrinol..

[189] Piper T., Geyer H., Haenelt N., Huelsemann F., Schaenzer W., Thevis M. (2021). Current Insights into the Steroidal Module of the Athlete Biological Passport. Int. J. Sports Med..

[190] Otzel D.M., Nichols L., Conover C.F., Marangi S.A., Kura J.R., Iannaccone D.K., Clark D.J., Gregory C.M., Sonntag C.F., Wokhlu A. (2024). Musculoskeletal and body composition response to high-dose testosterone with finasteride after chronic incomplete spinal cord injury—A randomized, double-blind, and placebo-controlled pilot study. Front. Neurol..

[191] Watson C.J., Stone G.L., Overbeek D.L., Chiba T., Burns M.M. (2022). Performance-enhancing drugs and the Olympics. J. Intern. Med..

[192] Thevis M., Geyer H., Mareck U., Flenker U., Schänzer W. (2007). Doping-control analysis of the 5alpha-reductase inhibitor finasteride: Determination of its influence on urinary steroid profiles and detection of its major urinary metabolite. Ther. Drug Monit..

[193] Iannella L., Colamonici C., Curcio D., Botrè F., de la Torre X. (2021). 5α-reductase inhibitors: Evaluation of their potential confounding effect on GC-C-IRMS doping analysis. Drug Test. Anal..

[194] Mazzarino M., Martellone L., Comunità F., de la Torre X., Molaioni F., Botrè F. (2019). Detection of 5α-reductase inhibitors by UPLC–MS/MS: Application to the definition of the excretion profile of dutasteride in urine. Drug Test. Anal..

[195] Oleksak P., Nepovimova E., Valko M., Alwasel S., Alomar S., Kuca K. (2024). Comprehensive analysis of prohibited substances and methods in sports: Unveiling trends, pharmacokinetics, and WADA evolution. Envirom. Toxicol. Pharmacol..

[196] Pieters H.C., Green E., Sleven M., Stanton A.L. (2020). Aromatase inhibitors: The unexpected breast cancer treatment. J. Ger. Oncol..

[197] Althuwaibi M.F., Fernandez-Garcia C., Hayes L., McNally R., Coughlan D. (2023). Systematic review of economic evaluations of aromatase inhibitors in estrogen receptor-positive breast cancer: Quality evaluation. BMC Health Serv. Res..

[198] Hyder T., Marino C.C., Ahmad S., Nasrazadani A., Brufsky A.M. (2021). Aromatase Inhibitor-Associated Musculoskeletal Syndrome: Understanding Mechanisms and Management. Front. Endocrinol..

[199] Rochoy M., Danel A., Chazard E., Gautier S., Berkhout C. (2022). Doping with aromatase inhibitors and oestrogen receptor modulators in steroid users: Analysis of a forum to identify dosages, durations and adverse drug reactions. Therapies.

[200] Favretto D., Snenghi R., Pertile R., El Mazloum R., Tucci M., Visentin S., Vogliardi S. (2019). Hair analysis to discriminate voluntary doping vs inadvertent ingestion of the aromatase inhibitor letrozole. Drug Test. Anal..

[201] Wu Y.C., Sung W.W. (2024). Clomiphene Citrate Treatment as an Alternative Therapeutic Approach for Male Hypogonadism: Mechanisms and Clinical Implications. Pharmaceuticals.

[202] Shen J., He Y., Li S., Chen H. (2024). Crosstalk of methylation and tamoxifen in breast cancer (Review). Mol. Med. Rep..

[203] Fohlin H., Nordenskjöld A., Rosell J., Fernö M., Fornander T., Rydén L., Skoog L., Nordenskjöld B., Stål O. (2024). Breast cancer hormone receptor levels and benefit from adjuvant tamoxifen in a randomized trial with long-term follow-up. Acta Oncol..

[204] Vora D., Dandekar A., Bhattaccharjee S., Singh O.N., Agrahari V., Peet M.M., Doncel G.F., Banga A.K. (2022). Formulation Development for Transdermal Delivery of Raloxifene, a Chemoprophylactic Agent against Breast Cancer. Pharmaceutics.

[205] Alwashmi A.S.S., Khan N.U., Chen T. (2025). Risk-benefits assessment of tamoxifen or raloxifene as chemoprevention for risk reduction of breast cancer among BRCA1 and BRCA2 carriers: A meta-analysis. Sci. Rep..

[206] Kim N., Lukong K.E. (2025). Treating ER-positive breast cancer: A review of the current FDA-approved SERMs and SERDs and their mechanisms of action. Oncol. Rev..

[207] Miller G.D., Moore C., Nair V., Hill B., Willick S.E., Rogol A.D., Eichner D. (2019). Hypothalamic-Pituitary-Testicular Axis Effects and Urinary Detection Following Clomiphene Administration in Males. J. Clin. Endocrinol. Metab..

[208] Havnes I.A., BordadoHenriksen H.C., Johansen P.W., Bjørnebekk A., Neupane S.P., Hisdal J., Seljeflot I., Wisløff C., Jørstad M.L., McVeigh J. (2024). Off-label use of clomiphene citrate to treat anabolic androgenic steroid induced hypogonadism upon cessation among men (CloTASH)—A pilot study protocol. MethodsX.

[209] García-Rodríguez C., Mujica P., Illanes-González J., López A., Vargas C., Sáez J.C., González-Jamett A., Ardiles Á.O. (2023). Probenecid, an Old Drug with Potential New Uses for Central Nervous System Disorders and Neuroinflammation. Biomedicines.

[210] Hou Z., Ma A., Mao J., Song D., Zhao X. (2023). Overview of the pharmacokinetics and pharmacodynamics of URAT1 inhibitors for the treatment of hyperuricemia and gout. Expert. Opin. Drug Metab. Toxicol..

[211] Abdel-Fattah M.M., Abo-El Fetoh M.E., Afify H., Ramadan L.A.A., Mohamed W.R. (2023). Probenecid ameliorates testosterone-induced benign prostatic hyperplasia: Implications of PGE-2 on ADAM-17/EGFR/ERK1/2 signaling cascade. J. Biochem. Mol. Toxicol..

[212] Wilson R.C., Arkell P., Riezk A., Gilchrist M., Wheeler G., Hope W., Holmes A.H., Rawson T.M. (2022). Addition of probenecid to oral β-lactam antibiotics: A systematic review and meta-analysis. J. Antimicrob. Chemother..

[213] Everts R.J., Gardiner S.J., Zhang M., Begg R., Chambers S.T., Turnidge J., Begg E.J. (2021). Probenecid effects on cephalexin pharmacokinetics and pharmacodynamics in healthy volunteers. J. Infect..

[214] Huriez P., Ourghanlian C., Razazi K., Vindrios W., Hulin A., Lepeule R., Habibi A., Gallien S. (2022). Probenecid, an old β-lactams pharmacokinetic enhancer for a renewed use: A retrospective study. Infect. Dis. Now..

[215] Ventura R., Segura J. (2010). Masking and manipulation. Handb. Exp. Pharmacol..

[216] Bird S.R., Goebel C., Burke L.M., Greaves R.F. (2016). Doping in sport and exercise: Anabolic, ergogenic, health and clinical issues. Ann. Clin. Biochem..

[217] Hemmersbach P. (2020). The Probenecid-story—A success in the fight against doping through out-of-competition testing. Drug Test. Anal..

[218] Jaque-Fernandez F., Allard B., Monteiro L., Lafoux A., Huchet C., Jaimovich E., Berthier C., Jacquemond V. (2023). Probenecid affects muscle Ca^2+^ homeostasis and contraction independently from pannexin channel block. J. Gen. Physiol..

[219] Cadwallader A.B., De La Torre X., Tieri A., Botrè F. (2010). The abuse of diuretics as performance-enhancing drugs and masking agents in sport doping: Pharmacology, toxicology and analysis. Br. J. Pharmacol..

[220] De Wilde L., Roels K., Polet M., Van Eenoo P., Deventer K. (2018). Identification and confirmation of diuretics and masking agents in urine by turbulent flow online solid-phase extraction coupled with liquid chromatography-triple quadrupole mass spectrometry for doping control. J. Chromatogr. A.

[221] Moscovici H.F., Lara P.H.S., Solera F.A.G., Cohen M., Pagura J.R., Arliani G.G. (2024). Doping control in male soccer players in brazil: 10 years of follow-up. ActaOrtop. Bras..

[222] Lee T.W., Bae E., Hwang K., Jang H.N., Park H.J., Jeon D.H., Cho H.S., Chang S.H., Park D.J. (2017). Severe hypokalemic paralysis and rhabdomyolysis occurring after binge eating in a young bodybuilder: Case report. Medicine.

[223] Bondaryk M., Kurzątkowski W., Staniszewska M. (2013). Antifungal agents commonly used in the superficial and mucosal candidiasis treatment: Mode of action and resistance development. Adv. Dermatol. Allergol..

[224] Pivonello R., Ferrigno R., De Martino M.C., Simeoli C., Di Paola N., Pivonello C., Barba L., Negri M., De Angelis C., Colao A. (2020). Medical Treatment of Cushing’s Disease: An Overview of the Current and Recent Clinical Trials. Front. Endocrinol..

[225] Palermo A., Botrè F., de la Torre X., Fiacco I., Iannone M., Mazzarino M. (2016). Drug-drug interactions and masking effects in sport doping: Influence of miconazole administration on the urinary concentrations of endogenous anabolic steroids. Forensic Toxicol..

[226] Mazzarino M., Comunità F., de la Torre X., Molaioni F., Botrè F. (2021). Effects of the administration of miconazole by different routes on the biomarkers of the “steroidal module” of the Athlete Biological Passport. Drug Test. Anal..

[227] Sessa F., Salerno M., Di Mizio G., Bertozzi G., Messina G., Tomaiuolo B., Pisanelli D., Maglietta F., Ricci P., Pomara C. (2018). Anabolic Androgenic Steroids: Searching New Molecular Biomarkers. Front. Pharmacol..

[228] Sessa F., Maglietta F., Bertozzi G., Salerno M., Di Mizio G., Messina G., Montana A., Ricci P., Pomara C. (2019). Human Brain Injury and miRNAs: An Experimental Study. Int. J. Mol. Sci..

[229] Sessa F., Salerno M., Cipolloni L., Bertozzi G., Messina G., Mizio G.D., Asmundo A., Pomara C. (2020). Anabolic-androgenic steroids and brain injury: miRNA evaluation in users compared to cocaine abusers and elderly people. Aging.

[230] Sessa F., Salerno M., Bertozzi G., Cipolloni L., Messina G., Aromatario M., Polo L., Turillazzi E., Pomara C. (2020). miRNAs as Novel Biomarkers of Chronic Kidney Injury in Anabolic-Androgenic Steroid Users: An Experimental Study. Front. Pharmacol..

[231] Shirpoor A., Naderi R. (2024). Nandrolone decanoate induced kidney injury through miRNA-146a targeting IRAK1 and TRAF6 via activation of the NF-κB pathway: The effect of moderate exercise. Steroids.

[232] Martín-Escudero P., Muñoz-Guerra J.A., García-Tenorio S.V., Serrano-Garde E., Soldevilla-Navarro A.B., Cortes-Carrillo N., Galindo-Canales M., del Prado N., Fuentes-Ferrer M., Fernández-Pérez C. (2021). Bioanalytical Detection of Steroid Abuse in Sports Based on the Androgenic Activity Measurement. Chemosensors.

[233] Coll S., Shiomura S., Alechaga É., Bressan C., Monfort N., Okano M., Ventura R. (2025). Detection of Oral Testosterone Undecanoate Administration in UGT2B17 del/del and del/ins Individuals. Part II: Urinary Endogenous Steroid Sulfate Markers. Drug Test. Anal..

[234] Okano M., Shiomura S. (2024). Effectiveness of blood steroidal passport markers for detecting testosterone abuse in Asians. Drug Test. Anal..

